# Thermoneutrality and severe malaria: investigating the effect of warmer environmental temperatures on the inflammatory response and disease progression

**DOI:** 10.3389/fimmu.2023.1128466

**Published:** 2023-06-07

**Authors:** Fiorella Vialard, Isabelle Allaeys, George Dong, Minh Phuong Phan, Urvashi Singh, Marie Josée Hébert, Mélanie Dieudé, David Langlais, Eric Boilard, David P. Labbé, Martin Olivier

**Affiliations:** ^1^ Infectious Diseases and Immunity in Global Health, Research Institute of the McGill University Health Centre, Montréal, QC, Canada; ^2^ Department of Microbiology and Immunology, McGill University, Montreal, QC, Canada; ^3^ Centre Hospitalier Universitaire de Québec, Université Laval, Québec, QC, Canada; ^4^ Department of Human Genetics, McGill University Genome Centre, Faculty of Medicine, McGill University, Montreal, QC, Canada; ^5^ Centre de Recherche, Centre Hospitalier de l’Université de Montréal, Montréal, QC, Canada; ^6^ Département Microbiologie, Infectiologie et Immunologie, Faculté de Médecine, Université de Montréal, Montréal, QC, Canada; ^7^ Division of Urology, Department of Surgery, McGill University, Montréal, QC, Canada

**Keywords:** thermoneutrality, malaria, *Leishmania*, extracellular vesicles, *Plasmodium berghei* ANKA, *Leishmania major*, inflammatory response, disease progression

## Abstract

**Introduction:**

Most studies using murine disease models are conducted at housing temperatures (20 – 22°C) that are sub-optimal (ST) for mice, eliciting changes in metabolism and response to disease. Experiments performed at a thermoneutral temperature (TT; 28 – 31°C) have revealed an altered immune response to pathogens and experimental treatments in murine disease model that have implications for their translation to clinical research. How such conditions affect the inflammatory response to infection with Plasmodium berghei ANKA (PbA) and disease progression is unknown. We hypothesized that changes in environmental temperature modulate immune cells and modify host response to malaria disease. To test this hypothesis, we conducted experiments to determine: (1) the inflammatory response to malarial agents injection in a peritonitis model and (2) disease progression in PbA-infected mice at TT compared to ST.

**Methods:**

In one study, acclimatized mice were injected intraperitoneally with native hemozoin (nHZ) or Leishmania at TT (28 – 31°C) or ST, and immune cells, cytokine, and extracellular vesicle (EV) profiles were determined from the peritoneal cavity (PEC) fluid. In another study, PbA-infected mice were monitored until end-point (i.e. experimental malaria score ≥4).

**Results:**

We found that *Leishmania* injection resulted in decreased cell recruitment and higher phagocytosis of nHZ in mice housed at TT. We found 398 upregulated and 293 downregulated proinflammatory genes in mice injected with nHZ, at both temperatures. We report the presence of host-derived EVs never reported before in a murine parasitic murine model at both temperatures. We observed metabolic changes in mice housed at TT, but these did not result to noticeable changes in disease progression compared to ST.

**Discussion:**

To our knowledge, these experiments are the first to investigate the effect of thermoneutrality on a malaria murine model. We found important metabolic difference in mice housed at TT. Our results offer insights on how thermoneutrality might impact a severe malaria murine model and directions for more targeted investigations.

## 1 Introduction

Malaria is a tropical disease caused by blood-apicomplexan parasites of the *Plasmodium* genus and transmitted by the bite of infected female *Anopheles* spp. mosquitos (1). The World Health Organization (WHO) attributed 247 million human cases and 619 thousand deaths to malaria in 2021 in endemic regions (1). This constituted an increase of 21 million cases since 2019, mostly attributed to the wide disruption to testing services and treatment available in sub-Saharan Africa due to the COVID-19 pandemic (1). These deaths typically occur in non-immune individuals (e.g. children or adults from low-transmission/non-endemic areas where exposure is low) in which the clinical presentation of the disease progresses to severe malaria (2). Severe malaria is most often caused by *Plasmodium falciparum* and is characterized by high parasitemia and organ pathologies (e.g. hepato-splenomegaly, respiratory distress and kidney failure) resulting from parasite sequestration to these organs followed by immune-mediated cytotoxicity (2, 3). One of the hallmarks of severe malaria due to *P. falciparum* infection is cerebral malaria (CM), during which patients experience coma-like symptoms following parasite-sequestration to the brain (3).

Despite advances in programs targeting malaria elimination, the milestone set by the WHO to reduce mortality by at least 40% by 2020 was not achieved and the 2025 milestone to reduce it by at least 75% may not be met if drastic actions are not taken (1). Several factors have been identified for the stagnation in elimination efforts: the COVID-19 epidemic, parasite resistance to anti-malarial drugs, vector resistance to long-lasting insecticide-treated nets, the rise of *Plasmodium* mutants undetectable with rapid diagnostic tests, and climate change (1, 4). The WHO has recommended continued investment in malaria research and development initiatives in order to achieve elimination of the disease (1). Therefore, a more thorough understanding of the effect of the parasite on the immune system of the vertebrate host is critical to future control of the disease.

The erythrocytic cycle of *Plasmodium* spp. is key to understanding malarial disease because it is the stage of the parasitic life-cycle in which most symptoms occur (5). Briefly, after the vector transmits *Plasmodium* spp. sporozoites to the vertebrate host and the parasites undergo a round of replication in the liver, the resulting merozoites initiate the blood stage by invading red blood cells (RBCs), developing asexually to trophozoites, and forming schizonts that eventually rupture the infected RBCs (iRBCs) and release additional merozoites (6). Each merozoite can then infect a naïve RBC and initiate multiple rounds of RBC infection and merozoite release (6). A fraction of trophozoites differentiate and form gametocytes that can reproduce sexually in the mosquito host (6). The lysis of iRBCs results in the release of parasites and iRBC cytosolic content, both of which can interact with and affect innate immune cell function (6). The engaged immune cells release inflammatory factors, such as pro-inflammatory cytokines/chemokines (e.g. IL-1β, TNF-α, Il-12, IL-10, Il-6), nitric oxide (NO) and reactive oxygen species (ROS) that drive the inflammatory response and lead to the fever-like symptoms observed in malaria patients (6, 7). One of the cytosolic iRBC contents released during schizont lysis is native hemozoin (nHZ), an inorganic crystal produced from the digestion of hemoglobin by the parasite within iRBCs (6). It is also referred to as malarial pigment because it confers a brown color to blood and organs where it tends to accumulate in patients with severe malaria suffering from hyper-parasitemia (6). Its effect on the malarial inflammatory response of the host has been extensively characterized in our lab using *in vivo* models (6, 8). Upon interaction or engulfment by various immune cells (i.e. monocytes, macrophages, and, neutrophils), nHZ induces the expression of chemokines (i.e. MIP-1α/CCL3, MIP-1β/CCL4, MIP-2/CXCL2, and MCP-1/CCL2) chemokine receptors (i.e. CCR1, CCR2, CCR5, CXCR2, and CXCR4), cytokines (i.e. IL-1β and IL-6); and myeloid-related proteins (i.e. S100A8, S100A9, and S100A8/A9) (6, 8).

Extracellular vesicles (EVs) are cell-membrane derived nanoparticles released by all living cells and that can transport nucleic acids, proteins, and lipids from cell to cell and elicit biological responses. In the context of malaria, there is evidence that EVs released by iRBCs and other host cells can contribute to the severity of the disease by modulating the inflammatory response (9–13).

Mouse models of disease have been used effectively to study malaria inflammatory response and disease progression. *Plasmodium berghei* ANKA (PbA)-infected C57BL/6 mice are used as a model for severe malaria because it mimics most of the symptoms occurring in human patients (14–16). In addition, a mouse peritonitis model has been used to study the inflammatory response to several parasitic agents.

Another important group of neglected tropical diseases is leishmaniasis caused by other apicomplexan parasites that are also transmitted by the bite of an infected arthropod vector, i.e., the sandfly (17). *Leishmania* spp. parasites have also been shown to induce an inflammatory response in the mammal host and can grow in high numbers in culture (18–20). As such, they can be used as a strong positive control in peritonitis model experiments. Our lab and others have shown that injecting mice intraperitoneally (IP) with the *Leishmania* parasite causes the recruitment of inflammatory cells to the peritoneal cavity (PEC) (18, 19). However, the major metabolic limitations of this murine model and others should be considered as it has been implicated in the lack of translatability from mouse model studies to human clinical trials (21).

Thermoneutral temperature (TT) is the environmental temperature at which an organism does not have to use energy to generate or lose heat. In mice, this temperature is around 30°C whereas in humans it is closer to 20°C (21). Since humans spend the majority of their time at those temperatures, they are in a metabolic state of thermoneutrality. However, almost all studies using mice are conducted at temperatures below their TT (i.e., 30°C) (21). Housing mice at sub-optimal temperature (ST; i.e., 20°C) as a standard housing temperature has been chosen for a number of reasons (e.g. comfort of animal facility staff, ammonia-level control and cost) that are unrelated to experimental optimization. ST can impact the inflammatory response because the energy required for immune cell metabolism is instead redirected towards non-shivering thermogenesis (21). The difference between the human and research model metabolic state has been implicated in low translatability of research projects; its effect has been studied in the context of cancer, metabolic diseases, respiratory diseases, graft-vs.-host disease, cardiovascular diseases, Alzheimer’s disease, bacterial and viral diseases. However, reports on parasitic infections at thermoneutrality are limited (21). The inflammatory response to parasitic infection and progression of disease might be exacerbated at TT if the immune cells are more performant and induce a striking response. Alternatively, they may be reduced if the animal’s immune cells are able to control the parasitic infection without causing severe pathologies.

Therefore, we hypothesized that changes in environmental temperature modulate immune cells and modify host response to malaria disease. To test this hypothesis, we developed two objectives to determine (1): the inflammatory response to nHZ injection in a peritonitis model and (2) disease progression in PbA-infected mice at TT compared to ST.

## 2 Material and methods

### 2.1 Animals and ethics

All experiments using mice were carried out in pathogen-free housing under the Animal Use Protocol 7607 at McGill University, according to Canadian Council on Animal Care (CACC) Guidelines and approved by the McGill University Animal Care Committee.

Female Balb/c wild-type mice (5-7 weeks) were purchased from Charles River Laboratories (Wilmington, MA, US) acclimatized for 1 week in standard housing, and used to obtain our preliminary results of sHZ-mediated inflammation using a peritonitis model.

Female C57BL/6 wild-type mice (6-8 weeks) from our in-house colony were used for PbA infection to obtain nHZ in standard housing conditions.

For all other experiments, female C57BL/6 wild-type mice (3-4 weeks old) were purchased from Charles River Laboratories and acclimatized in standard housing at ST (19-22°C) or in a temperature-controlled cabinet at TT (27-31°C) for three weeks before the start of experiments. We chose younger mice for this trial so that after their acclimatization period, they would reach a standardized age for our malaria experiments described here and previously (8, 22). Mice were housed individually to prevent warming by clustering in ST-housed mice, as is standard in thermoneutrality research (21, 23–25). The cabinet used to house the mice at TT could only ever reach 1-3°C above the room temperature. Therefore, we ensured that the environmental room temperatures were oscillating between 21.5°C at night and 26.7°C, during the day to account for the fact that the activity levels of the mice (and thus the temperature inside the cage) increases at night, increasing the temperature higher than the 3 additional degrees provided by the cabinet (21). We ensured that mice in each group were exposed to their assigned environmental temperatures by monitoring cage temperatures twice a week at different times of the day. We provided a representative example of room (**Supplementary Figure 1**) and cage temperatures (**Supplementary Table 1**). Cages were changed once a week (i.e. Mondays) and humidity level measured by humidity sensors in the cabinet, ammonia level measured by color-based sensors in each cage, food/water intake measured by weighing the remaining amount in feeder/bottle, and mouse body weights measured by an electronic scale, were monitored twice a week (Mondays and Fridays or Tuesdays and Thursdays) at different times of the day.

### 2.2 Native hemozoin preparation

C57BL/6 mice were treated with iron dextran before infection with PbA following a previously established protocol (22). This treatment enabled us to obtain blood samples with high parasitemia by preventing experimental cerebral malaria (ECM) that normally occurs in PbA-infected mice (22). The spleens of two infected mice (parasitemia > 60%) were collected and homogenized for nHZ extraction following a previously established protocol (26). Briefly, the homogenates were treated with Tris-HCl (100mM) and centrifuged at 1721xg for 10 min to release and separate the nHZ crystals from cells. The resulting pellet containing nHZ crystals was washed in PBS and centrifuged three times at 1721xg before drying on a heat plate at 95°C overnight. The dried nHZ was weighed, resuspended in PBS, and sonicated on ice for 15 min at maximum amplitude using an Ultrasonic Liquid Processor s4000 (Misonix, Farmingdale, NY, USA).

### 2.3 Synthetic hemozoin preparation

The synthesized hemozoin powder was prepared, as described in a previous publication (26). Subsequently, 50 mg of sHz powder were solubilized in 5 ml of 1X PBS to achieve the final concentration of 10 mg/ml. The sample was sonicated for 2 min at the lowest amplitude using an Ultrasonic Liquid Processor s4000 (Misonix) for resuspension and aliquots of 200 μl were stored at -20°C in amber Eppendorf tubes.

### 2.4 Parasite cultures

*Leishmania major* strain NIH S (MHOM/SN/74/Seidman) clone A2, was cultured at 25°C in Schneider’s Drosophila Medium (Gibco-BRL, Grand Island, NY) supplemented with 10% heat-inactivated FBS, 5mg/ml HEMIN, 2 mM L-glutamine, 100 U/ml penicillin, and 100 μl/ml streptomycin. Cultures of promastigotes growing at logarithmic phase (day 3-4 post passage) were passaged biweekly at least twice and grown to stationary phase (day 6-8 post passage) for use in mouse studies (20).

### 2.5 Intraperitoneal inoculation

For our preliminary inflammatory response trial at ST, sHZ was thawed and diluted in 1X PBS into 3 doses: 100 μg, 250 μg, 500 μg of sHZ in a final volume of 200 μl. Control mice were injected with 200 μl of PBS.

For all other IP injections, frozen nHZ was thawed and diluted in 1X PBS to a final concentration of 1 mg in 200 μl (5mg/mL). Mice at ST and TT were injected IP with 200 μl of endotoxin-free PBS (negative control; n=3), nHZ (n=3) or 10^8^ *L. major* promastigotes (positive control; n=3) in 2 separate experiments.

For both IP injection trials, after six hours, 5 ml of cold PBS were injected into the peritoneal cavity (PEC) and immune cells were collected by lavage. For the preliminary trial, a total of 32 mice were injected (n=8/experimental group). In the other, a total of 18 samples were recovered (n=6/experimental group) for each temperature (total n = 36).

### 2.6 Inflammatory cells monitoring and bio-banking for further analysis

The number of live cells in the PEC lavage samples was counted with a hemocytometer as follows. An aliquot of 150 μL of the cell suspension was transferred to a Cytofunnel (ThermoFisher, Waltham, MA, USA) and centrifuged at 10xg in a Shandon Cytospin 4 centrifuge for 5 min for deposition onto a glass slide (ThermoFisher). The slides were dried and stained using a Diff-Quick kit following the manufacturer’s protocol (RAL Diagnostics, Martillac, France). Cells were phenotyped by microscopy at 100X with oil-immersion. The slides were incubated for 24 and 48 hours at 37°C, dried and stained using a Diff-Quick kit to visualize phagocytosis of *L. major* by macrophages and neutrophils. The remaining volumes of the PEC lavage samples were centrifuged at 297xg for 10 min to separate cells and supernatants. Supernatants were aliquoted and frozen at -80°C for further analyses. Cell pellets were resuspended in 1mL Trizol (ThermoFisher) for 5 min and frozen at -80°C for further analyses.

### 2.7 Extracellular vesicle extraction

Aliquots of approximately 2 mL of frozen supernatants from PEC lavage were thawed, pooled (2 or 3 mice per experimental group for each temperature in the thermoneutrality trials and 4 mice/group in the preliminary trial) and filtered using a 0.45 μm filter to exclude debris. The filtrate was transferred to 17 mL thin-wall polypropylene tubes (Beckman Coulter, Brea, CA, USA) and completed with EV buffer (137mM NaCl, 20mM HEPES). The tubes were centrifuged at 100000xg (RCFavg) overnight (18 hours) at 4°C in an SW32.1 Ti swinging bucket rotor (Beckman Coulter). The supernatant was discarded, fresh exosome buffer was added to the tube to wash the pellet and centrifuged using the same conditions. This wash step was repeated, and the pellet was re-suspended in 400ul of EV buffer. Protein concentrations were determined using a microBCA assay following the manufacturer’s protocol (ThermoFisher). The results were read in a Tecan plate reader. The remainder of the extracted EVs were aliquoted and frozen at -80°C for further analyses. For each of the thermoneutrality trials, the whole process was repeated a second time with the PEC lavages of the second round of injections.

### 2.8 Transmission electron microscopy

EVs suspended in exosome buffer for each of the PEC lavages (n=4 in preliminary trial and n= 6 in the thermoneutrality trial) were deposited onto Fomvar carbon grids (Mecalab, Montreal, QC, Canada), fixed with 1% glutaraldehyde in 0.1M sodium cacodylate buffer, washed 3 times with autoclaved Milli-Q water, and stained with 1% uranyl acetate. Each step was performed for 1 min in duration. Samples were visualized with a FEI Technai-12 120kV transmission electron microscope and AMT XR80C CCD Camera (Facility for Electron Microscopy Research, McGill University, Montreal, Canada).

### 2.9 Nanoparticle tracking analysis

Aliquots of extracted EV (80 μl) were diluted in 1000 μl of exosome buffer and injected into the sample chamber for each of the PEC lavages (n=4 in preliminary trial and n=6 in the thermoneutrality trial). EV size distribution was determined using nanoparticle tracking analysis (NTA) software and a NanoSight NS300 instrument (Malvern Panalytical, Malvern, Worcestershire, UK). Three videos were captured for 30 seconds each at 37°C, using optimized camera settings that were kept consistent for all samples. Particle concentration, mean, median, and mode size were calculated and graphed using the NTA 3.4 Build 3.4.4 software (27).

### 2.10 High sensitivity flow-cytometry analysis

Aliquots of supernatant of 36 PEC lavage samples were analyzed through a method of high-sensitivity flow cytometry (hsFC) to phenotype the EV released by immune cells in these experiments. These analyses were performed using a BD FACSCanto II instrument (BD Biosciences) with forward scatter coupled to a photomultiplier tube small particles option (FSC-PMT) as described previously (28, 29). Size gating was determined using silica beads (Fluorescent green plain silica particles, 3PSI-G, Kisker Biotech, Steinfurt, Germany) of 100 nm, 500 nm and 1000 nm diameter. Data were analyzed using Diva (version 6.1.3) or FlowJo software (version 9.9.6). For quantitative analysis a known concentration of fluorescent counting beads (2 µm Cy5-silica particles (Nanocs Inc.) was added to each tube. 20 µl of mouse peritoneal lavage were labeled with 250 nM LWA300 proteasome probe (gift from Dr Marie-Josée Hébert), in a total volume of 50 μL annexin buffer for 1 h at 30°C (30, 31). Samples were then incubated with BV421-Annexin V (BD Biosciences) and 2 μM of CellTracker™ DeepRed (ThermoFisher Scientific) for 30 min at RT and then diluted into 300 µL of annexin buffer and analyzed by flow cytometry.

### 2.11 EV labeling

20 µl of each mouse peritoneal lavage was also labeled with V450 anti-mouse CD45 (clone 11F30, BD Biosciences), FITC anti-mouse CD41 (clone MWReg30, BD Biosciences), and BV421 anti-mouse Ly6G (clone 1A8, BD Biosciences) or MitoTrackerTM Deep Red FM for mitochondria (ThermoFisher Scientific) for 30 min at RT, diluted into 250 µL of PBS and analyzed by flow cytometry. Extracellular vesicles were first gated according to their fluorescence and then to their size and inner complexity (FSC PMT-H vs. SSC-H) to consider events between approximately 100 and 1000 nm. The staining specificity was confirmed by negative controls: 1) 0.2% Triton X-100 was used on positive samples for 20 min at RT, before labeling, 2) 20 mM EDTA were added to the annexin buffer prior to the Annexin V labeling. We also confirmed specificity with fluorescence minus one (FMO) and isotype control staining for each antibody used.

### 2.12 RNA sequencing (RNA-Seq) and analysis

*Sequencing*. Total RNA was extracted using RNeasy columns coupled with on-column DNase I digestion (Qiagen) from 36 PEC lavages. The RNA integrity was assessed on a TapeStation (Agilent, Santa Clara, CA, USA) followed by library preparation using the TruSeq ribodepleted mRNA stranded kit (Illumina, San Diego, CA, USA). Libraries were sequenced on a NovaSeq 6000 (Illumina) using a paired-end 100bp configuration aiming for 60x10^6^ paired rads per sample.

*Analysis.* The quality of sequence reads was assessed using the FastQC tool (Babraham Bioinformatics) (32). Overrepresented sequences (Illumina TruSeq Adapters) were removed by palindrome trimming with Trimmomatic V.0.36 (33). Low-quality bases (phred score < 33) were also removed. The high quality of trimmed sequence reads was validated by FastQC. The sequence reads were then mapped to the mouse *mm10* UCSC GRCm38 reference assembly using HISAT2 v2.2.0 with parameters -k 1 to avoid multi-mapped reads and –rna-strandness FR for strand-specific information (34). The files containing mapped reads were converted from SAM to BAM format and then sorted using Samtools (35). FastQC reports of trimmed sequence reads showed high frequency of duplicates in the samples, which were filtered out using PicardTools (MarkDuplicates) and then indexed using Samtools (36). Gene expression was quantified by counting the number of uniquely mapped reads using featureCounts with the -s parameter to perform strand-specific read counting (37). For data visualization, bigwigs were generated using genomeCoverageBed and wigToBigWig tools (UCSC, USA) (38, 39). Bigwigs were visualized using the Integrative Genomics Viewer (40). Bigwigs were scaled per million reads mapped to exons excluding the remaining rRNA reads. Reads mapped to rRNAs were removed to resolve any biases from incomplete ribodepletion. Genes with a minimum expression level of five counts per million (CPM) reads in at least 3 of the samples were retained. TMM normalization was applied with the edgeR Bioconductor package, and pairwise differential gene expression analyses were performed for the TT and ST samples comparing the endotoxin-free PBS with *L. major*-infected or nHz samples. One PBS-treated sample at TT was excluded from the analysis due to very low coverage. Genes with ≥|2| fold-change (FC) in expression and Benjamini-Hochberg adjusted p-values ≤ 0.001 were considered significant. Gene Ontology analysis was performed using g:Profiler (41). Plots were generated with the ggplot2 package in R (42).

### 2.13 Disease progression monitoring

Red blood cells infected with PbA parasites expressing a green fluorescent protein (GFP)-luciferase fusion protein (Malaria Research and Reference Reagent Resource Center, Manassas, BA, USA) were used for the disease progression study. Acclimatized mice were infected by IP inoculation of 10^4^ infected iRBC (n=13 or 16) or 200 ul of 1X PBS (n=3) as a mock control at each temperature. Starting from 3 days-post infection, tail-vein blood was collected daily. Blood smears were stained with Diff-Quik, and parasitemia was determined by counting at least 500 cells. Mouse body weight, food consumption, water intake, body temperature and clinical score were monitored three times a day, daily and averaged to obtain one measure per day. To determine percentage of changes in body mass attributed to PbA infection for each day, the following formula was used: (Weight of animal day x post-infection/weight of same animal day of infection) x 100. Body temperature was measured using an infrared thermometer pointed towards and 3-5 mm away from abdomen, as previously described (43). Clinical score was assessed as one point for each of the following ECM symptoms: ruffled fur, hunching, wobbly gait, limb paralysis, convulsions, and coma, as described previously (16). Mice were euthanized when they reached end-point, determined as clinical score ≥ 4 (between 7 and 10 days post-infection). Upon euthanasia, blood was collected by cardiac puncture and placed on ice. Spleens, livers, and lungs were collected, weighed and frozen in liquid nitrogen. Between 200 and 500 μl of blood was centrifuged at 367xg for 15 min. The serum was collected and frozen at -80°C.

### 2.14 Parasite load luciferase assay

Organs of infected mice acclimatized at ST (n=8) or TT (n=9) were thawed on ice and 10 to 40 μg was homogenized in 500 μl of lysis buffer (1X Tris-NaCl-EDTA, 20% Glycerol and 1% Igepal in ddH2O) with a sterile plastic homogenizer. Blood was homogenized in 100 μl lysis buffer. The homogenates were placed on a shaker in a cold room (4°C) overnight and then centrifuged at 206xg for 10 min. The supernatant was collected, and protein levels were determined using a Bradford assay following the manufacturer’s protocol (Bio-Rad, Mississauga, ON, Canada). Results were read in a Tecan Plate Reader. Organ and blood parasite loads were determined using the Firefly Luciferase kit following the manufacturer’s protocol (Biotum, Inc, Fremont, CA, USA). Results were read in a Synergy H4 luminescence reader. Parasite loads were determined by dividing the relative luminescence units (RLU) by protein amounts in the reading volume. The same procedure was followed for an infected and a non-infected mouse to validate this method.

### 2.15 Cytokine/chemokine analysis using multiplex array

Cytokine and chemokine levels were determined from 100 μl of cell culture supernatant from 18 lavages using a multiplex mouse cytokine array/chemokine array 10-plex assay (Eve Technologies, Calgary, AB, Canada) that provides detection for IFNγ, IL-1β, GM-CSF, IL-2, IL-3, IL-4, IL-6, IL-10, IL-12 (p70), MCP-1 and TNF-α (n=3/experimental group at each temperature).

Cytokine and chemokine levels were determined from 100 μl of 18 PEC lavage supernatant and 100 μl of 25 1:2 diluted serum samples using a multiplex mouse cytokine/chemokine array 32-plex assay (Eve Technologies) that provides detection for Eotaxin, G-CSF, GM-CSF, IFNγ, IL-1α, IL-1β, IL-2, IL-3, IL-4, IL-5, IL-6, IL-7, IL-9, IL-10, IL-12 (p40), IL-12 (p70), IL-13, IL-15, IL-17, IP-10, KC, LIF, LIX, MCP-1, M-CSF, MIG, MIP-1α, MIP-1β, MIP-2, RANTES, TNF-α, and VEGF.

### 2.16 Statistical analysis

All statistical analyses were conducted using Prism Software for Mac version 8.00 or later (Graphpad PrismTM, San Diego, CA, USA). All p-values were determined using two-way ANOVA or mixed effects model and Dunnet’s or Sidak’s multiple comparison test, as appropriate. Outliers were considered and removed only when there was a robust biological or manipulation error (i.e. mice not injected properly with IP treatment) reasoning.

## 3 Results

### 3.1 Hemozoin injection leads to an inflammatory response using a mouse model of peritonitis

The fever-like response observed in malarial patients occurs during the erythrocytic stage and is associated with the release of nHZ and other malarial agents from the iRBCs (6). To validate that HZ could indeed cause an inflammatory response in the PEC of mice under standard animal facility housing temperature (i.e. at ST), before starting our thermoneutrality trials, we injected groups of Balb/c mice with three different doses of sHZ to observe cell recruitment. We found that cells were being recruited to the PEC with the injection of sHZ compared to PBS but not necessarily in a dose-dependent manner (**Supplementary Figure 2A**). We examined the proportion of various immune cell populations and observed that the most abundant cells present in the PEC at baseline (i.e. PBS injection) were macrophages: over 80%, as expected (**Supplementary Figure 2B**) (44–47). The most abundant cell-type recruited after injection with sHZ were neutrophils: between 30 and 60% depending on the dose (**Supplementary Figure 2B**). The other cell-type proportions (i.e. basophils, eosinophils and lymphocytes) did not vary from baseline (**Supplementary Figure 2B**). The percentage of macrophages was reduced to 30% with the highest dose of sHZ baseline (**Supplementary Figure 2B**).

We evaluated whether sHZ would be phagocytized by macrophages and neutrophils isolated from the PEC of Balb/c mice injected with three doses of sHZ at ST by determining the percentage of cells containing sHZ. The phagocytic activity of macrophages (i.e., 25 – 40%) and neutrophils (i.e., 25 – 30%) varied depending on the dose of sHZ (**Supplementary Figure 3**). These experiments showed that HZ was able to cause an inflammatory response with associated cell recruitment and phagocytosis in a mouse model of peritonitis under standard animal facility ST conditions.

### 3.2 Recruitment of inflammatory cells to the site of injection is decreased at thermoneutral temperatures

**Figure 1:**
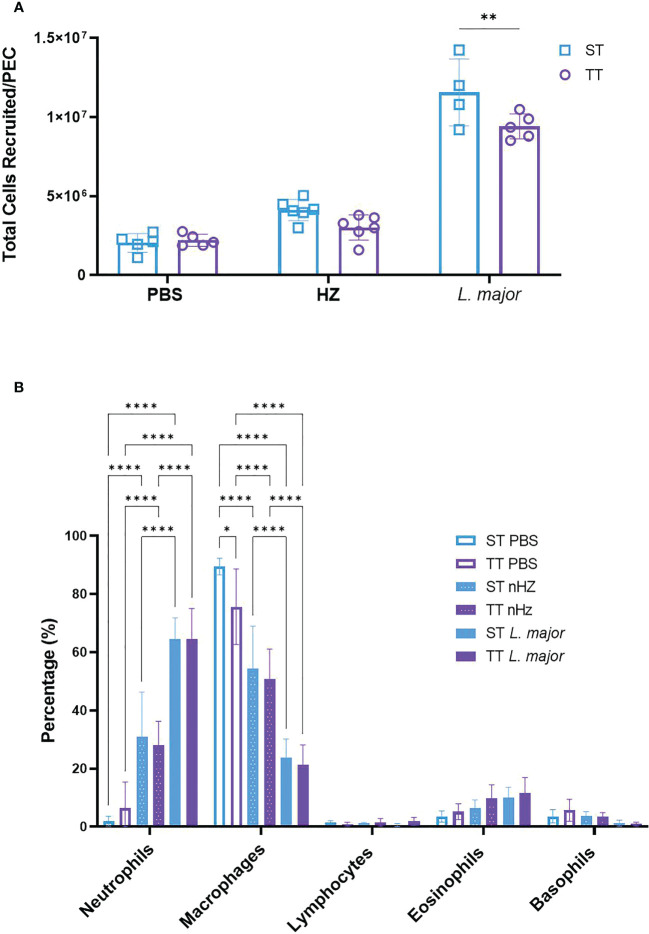
Recruitment of inflammatory cells following parasitic agent injection at thermoneutral (TT) and sub-optimal temperatures (ST). **(A)** Total number of inflammatory cells recruited and **(B)** proportions of immune cell subsets in the PEC of mice injected intraperitoneally with nHZ (1 mg) or 10^8^ *L. major* promastigotes housed at TT (n=5), or ST (n=5 or 6) was measured after 6 hours. Vertical bars represent mean +/- SEM. Two-way ANOVA followed by Sidak’s multiple comparison test was utilized. **** indicates p<0.0001, ** indicates p<0.01, * indicates a p<0.05. Only the significance bars showing a difference within a cell type are shown in **(B)**. Data is representative of 2 pooled independent experiments.

To determine whether the mouse inflammatory response to malarial or leishmanial components differed at TT compared to ST, we injected groups of mice with nHZ or *L. major* promastigotes intraperitoneally and collected PEC lavages after six hours. In these experiments, we used nHZ instead of sHZ because we had shown that the latter induces an inflammatory response *in vivo* but we wanted to validate our results with nHZ which is more specific to the species of *Plasmodium* we were using for our model (8). *L. major* was used as a positive parasitic control as its role in the inflammatory response has been documented (18, 19). We observed a lower recruitment of total inflammatory cells in the PEC of mice injected with *L. major* (i.e. approx. 600-cell increase) at TT compared to mice housed at ST (i.e. approx. 1000-cell increase) relative to PBS-treated mice at the corresponding temperature (**Figure 1A**). This trend was also observed for mice treated with nHZ but was not statistically significant (**Figure 1A**). The total recruited cells in nHZ-treated mice at ST were comparable to our previous results obtained with Balb/c mice (**Supplementary Figure 3**).

We examined the proportion of various immune cell populations and observed that the most abundant cells present in the PEC at baseline (i.e., PBS injection) were macrophages (89.4% at ST and 79.6% at TT) as expected from our experiments with sHZ-treated mice at ST (**Supplementary Figure 2B**). In contrast, neutrophils which were present at low levels at baseline: 1.9% at ST and 6.5% at TT, were the most abundant cell type recruited to the PEC after stimulation with nHZ (31.0% at ST and 27.9% at TT; **Figure 1B**). These percentages are comparable to our previous results at ST (**Supplementary Figure 2B**). Neutrophils were also the most abundant cells recruited to the PEC at ST and TT following *L. major* injection, but to a higher degree (64.4%) than with nHZ inoculation (**Figure 1B**). The percentage of macrophages was reduced to 54.4% and 50.4% with nHZ injection and 23.8% and 21.2% with *L. major* injection at ST and TT, respectively (see **Figure 1B**). Altogether, these results suggest mainly no difference in inflammatory response in mice exposed to parasitic agents at thermoneutral temperatures.

### 3.3 Thermoneutrality enhances macrophage phagocytic activity induced by nHZ, but not *L. major* promastigotes

**Figure 2:**
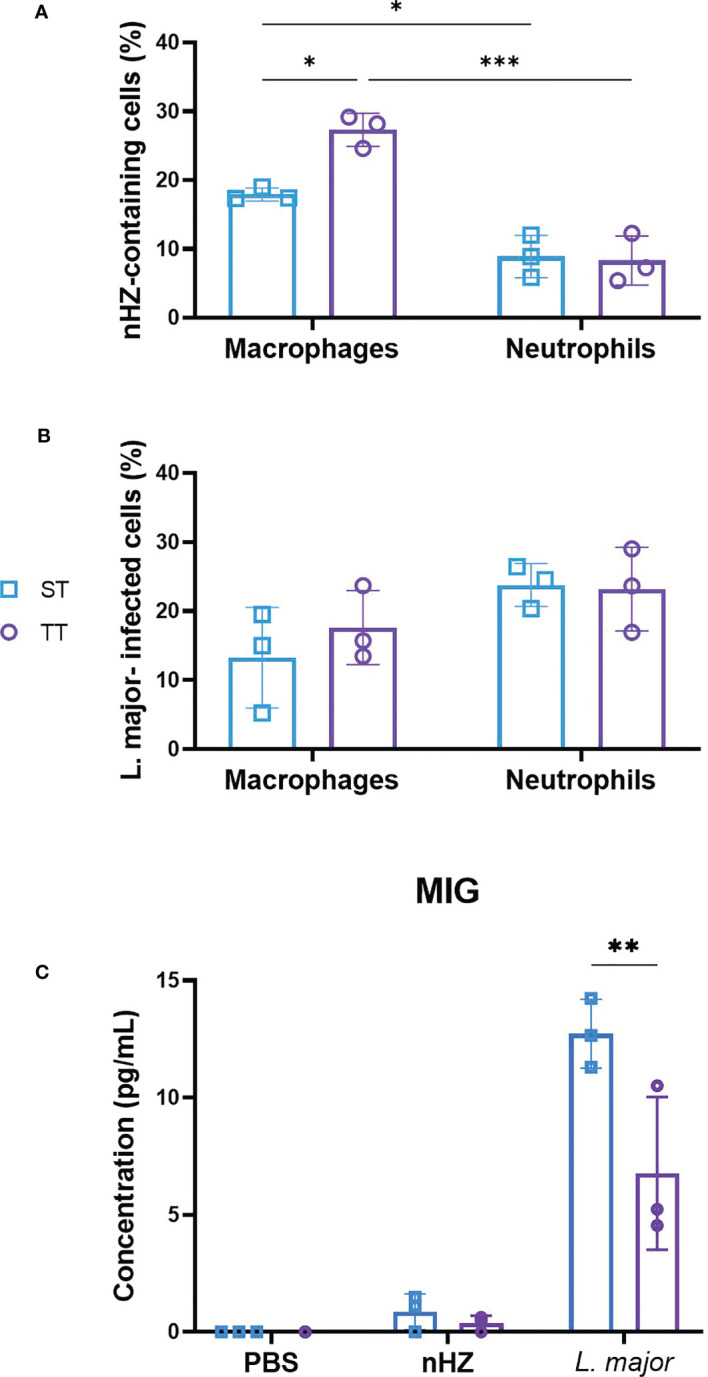
Thermoneutrality increases phagocytosis of native hemozoin (nHZ) but not *L. major* by macrophages. Percentages of macrophages and neutrophils containing **(A)** nHZ crystals or **(B)** *L. major* promastigotes were counted and **(C)** pro-inflammatory cytokines and chemokines concentrations were monitored in the PEC of mice injected intraperitoneally with 1 mg of nHZ or 10^8^ *L. major* promastigotes at sub-optimal (ST; n=3) and thermoneutral temperatures (TT; n=3). Apart from MIG **(C)** most post-inflammatory cytokines and chemokines did not differ in concentration at different temperatures. Vertical bars represent mean +/- SEM. Two-way ANOVA followed by Sidak’s multiple comparison test was utilized. *** indicates p<0.001, ** indicates p<0.01, * indicates a p<0.05. Significance bars between Macrophages ST and Neutrophils TT, and, Macrophage TT and Neutrophils ST in **(A)** are not shown for conciseness. Data representative of 1 out of 2 independent experiments.

We evaluated the phagocytic activity of macrophages and neutrophils isolated from the PEC of mice injected with parasitic agents at ST and TT by determining the percentage of cells containing nHZ or *L. major* promastigotes. Macrophages and neutrophils were involved in the phagocytosis of nHZ crystals at both temperatures in mice injected with 1 mg of nHZ (**Figure 2A**). We did not observe a difference in the phagocytic activity of neutrophils at the two temperatures (**Figure 2A**). However, the phagocytic activity of macrophages from mice housed at thermoneutrality was higher (27.3%) compared to those housed at ST (17.9%; **Figure 2A**). To determine if this was occurring in a cytokine-dependent manner we evaluated the levels of inflammatory cytokines and chemokines in the serum depending on housing temperature. MIG was the only pro-inflammatory chemokine that had significantly lower concentrations in mice housed at TT compared to those at ST (**Figure 2C**). This was consistent with other studies that have reported higher production of certain pro-inflammatory cytokines (e.g., MCP1, IFNγ, TNF-α, IL-6, and IL-1β) when exposed to ST (48–51). However, most cytokine/chemokine levels remained unchanged (**Supplementary Figure 4**) in accordance with results from previous studies in mice injected with lipopolysaccharide (LPS) at ST and TT (52). Others (e.g IL-1β, IL-10, and IFNγ) were sometimes present in levels too low to be detected by the multiplex array (i.e. fluorescence level below the lowest value required to measure concentration). Also, we did not observe a difference in phagocytic activity in macrophages from mice treated with *L. major* (**Figure 2B**). Overall, these data show that thermoneutrality may affect the macrophage phagocytic activity of malarial parasitic agents but we were not able to establish which cytokines were affected by these changes in our experiments.

### 3.4 Effect of thermoneutrality on profile of EVs released following parasitic agent injection

We sought to determine whether housing temperature influences EV release by immune cells after injection with parasitic agents by confirming their presence in biological samples through a conjunction of NTA and transmission electron microscopy (TEM) in concordance with the International Society for Extracellular Vesicles guidelines (53).

Because EVs have been shown to play an immunomodulatory role in parasitic diseases, we sought to determine whether injection with sHZ would lead to EV release (11, 12, 54–56). We confirmed the presence of EVs in biological samples with Nanoparticle Tracking Analysis (NTA). The EVs present in the samples ranged in size from 50 to 800 nm (**Supplementary Figure 5A**). These EVs were more abundant (10^9^ range) in the sizes between 100 to 180 nm in all samples (**Supplementary Figure 5B**). This experiment showed that HZ was able to cause increased EV release in a mice model of peritonitis under standard animal facility ST conditions.

**Figure 3:**
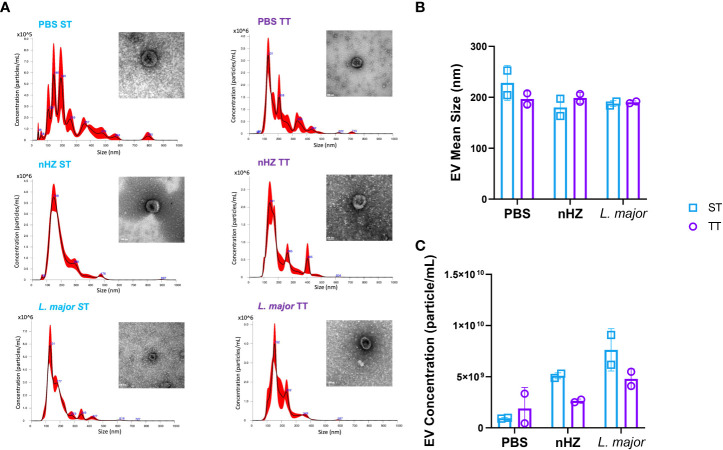
Extracellular vesicles (EVs) are released in the PEC of mice at thermoneutral (TT) and sub-optimal temperatures (ST). EV **(A)** distribution and morphologies, **(B)** mean size and **(C)** concentrations of mice injected intraperitoneally with 1mg of native hemozoin (nHZ) or 10^8^ *L. major* promastigotes. Mean size and concentrations were determined by nanoparticle tracking analysis and morphologies were visualized by transmission electron microscopy. Concentration in **(B)** were obtained by multiplying concentrations in **(A)** by the dilution factor used to prepare aliquots (80:1000 µl). Vertical bars represent mean +/- SEM. Two-way ANOVA followed by Sidak’s multiple comparison test was utilized. No temperature-based statistical difference was observed. Photos were taken at 49000x; scale bar represents 100nm. Data is representative of 2 EV extractions each corresponding to an independent experiment where 2 to 3 PEC lavages were pooled.

Transmission electron microscopy (TEM) images of the EVs isolated from the supernatant of peritoneal lavage fluid reveal that these EVs observed had similar morphology with their size up to 150nm in diameter. They are almost uniform in external shape, consisted of bilayer lipid membrane (**Figure 3A**).

NTA analysis revealed that the EVs present in the samples ranged in size from 50 to 800 nm (**Figure 3A**). A major peak at 100 to 180 nm was substantially more pronounced in the samples from mice injected with the parasitic agents (**Figure 3A**). Again, these results are concordant with results previously obtained with sHZ-infected mice at ST (**Supplementary Figure 5**). The EV profile differences did not appear to be housing-temperature dependent as mean size of EV were similar at TT and ST across injection groups (**Figure 3B**). However, the samples obtained from mice at TT showed a trend of lower concentrations of EVs compared to those obtained from mice at ST injected with nHZ or *L. major* (**Figure 3C**). These results are consistent with the overall decreased recruitment of inflammatory cells to the PEC.

**Figure 4:**
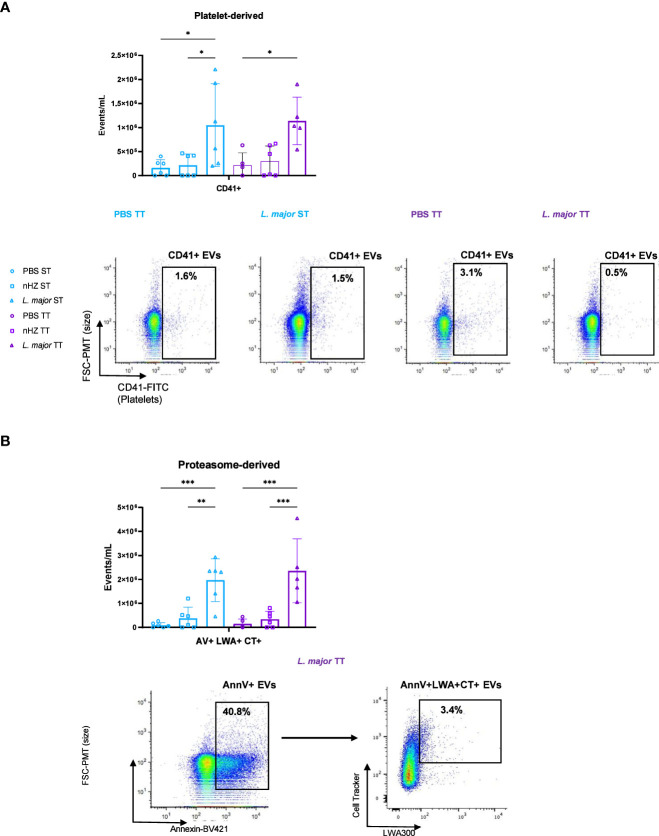
Subpopulations of extracellular vesicles (EVs) released following parasitic agent injections. **(A)** Platelet-derived (CD41+) and **(B)** Proteasome-derived (AV+LWA+CT+) EVs in the PEC of mice injected intraperitoneally with 1 mg of native hemozoin (nHZ) or 10^8^ *L. major* promastigotes were monitored by high-sensitivity flow cytometry at thermoneutral (TT; n=4, 5 or 6) or sub-optimal temperatures (ST; n =5 or 6). Vertical bars represent mean +/- SEM. Two-way ANOVA followed by Sidak’s multiple comparison test was utilized. *** indicate p<0.001, ** indicates p<0.01, * indicates a p<0.05. Significance bars between Control ST and *L. major* TT, nHZ ST and *L. major* TT, Control TT and *L. major* ST, and, nHZ TT and *L.major* ST in **(A)** and between Control ST and *L. major* TT, and, nHZ ST and *L.major* TT in **(B)** are not shown for conciseness. Bar charts representative of 2 pooled independent experiments and scatter plots illustrate results for single representative mice injected with PBS and *L. major*.

We employed a hsFC method to determine the level of EVs released and the cells from which they were released in the PEC of mice treated with nHZ or *L. major*. The identity of the cells from which EVs were released in the PEC was determined through detection of cell subtype-specific cell surface markers (28, 29). The total levels of EVs released in the PEC, as indicated by the AnnexinV marker, which is a phosphotidylserine (PS)-binding molecule that can be used to detect eukaryotic cell-derived EVs, did not vary between the different housing temperatures (**Supplementary Figure 6A**) (57). There was no difference in leukocyte-derived EV release at TT compared to ST (**Supplementary Figure 6B**). We investigated whether platelets were releasing EVs in the PEC which has not been shown in murine malaria or leishmaniasis but found no temperature-based differences (**Figure 4A**). The main population of cells recruited to the PEC by nHZ and *L. major* injection were neutrophils (**Figure 1B**). We therefore evaluated release of neutrophil-derived EVs bearing the Ly6G receptor in the PEC of mice but did not observe housing temperature-based differences (**Supplementary Figure 6C**). This is the first time that proteosome-derived EVs that are positive for the LWA+ proteasome marker are shown to be released in the PEC of mice injected *with L. major* but not in a housing-temperature dependent manner (**Figure 4B**) (58).

We conducted the same analysis with FMO and isotype control staining for each antibody used in the analysis to confirm specificity (**Supplementary Figure 7**). To further confirm that the small particles detected by hsFC were EVs, we treated representative aliquots of the PEC samples that had been previously stained with antibody-dyes with X-100 Triton and EDTA, which disrupt EV-membranes and surface PS, respectively. The treatments reduced EV detection in all samples, confirming that the particles detected by hsFC were EVs (**Supplementary Figure 8**). iRBC-derived EVs containing proteasomal factors have been shown to promote the growth of *P. falciparum* parasites previously but the exact role of the proteasomal derived host cell EVs has not been elucidated in parasitic diseases (59).

Overall, we did not observe any meaningful housing temperature-based difference in EVs released in the PEC of parasitic-agent treated mice.

### 3.5 Effect of thermoneutrality on cytokine expression

**Figure 5:**
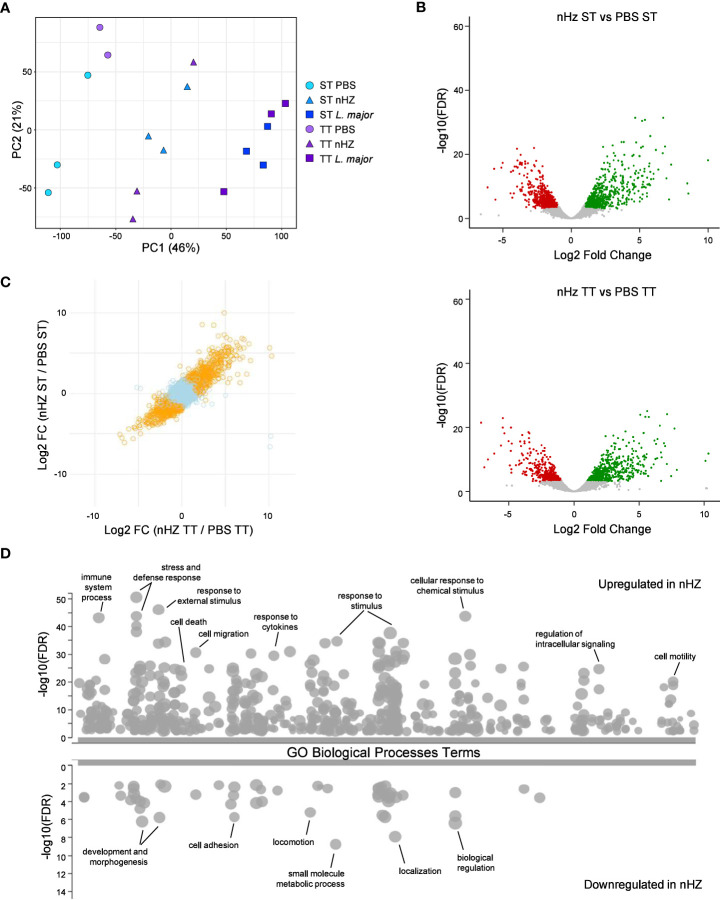
Transcriptome profiling of peritoneal cells following parasitic agent injections. The transcriptome of peritoneal cells from mice housed at sub-optimal (ST; n =5 or 6) or thermoneutral temperature (TT; n=5 or 6) and injected with either PBS, 1 mg of native hemozoin (nHZ), or 10^8^ *L. major* promastigotes was assessed by RNA-seq. **(A)** Principal component analysis showing that the type of parasitic agent injected is the major source of transcriptional variation. **(B)** Volcano plot for the differential expression analyses comparing the nHZ groups at ST and TT to their respective control PBS groups. The full DGE results are provided in **Supplementary Table 1**. **(C)** Plot comparing the fold change of gene expression in response to nHZ injection between ST and TT conditions. Orange dots represent genes with significant (FDR < 0.001) change in both ST and TT conditions. **(D)** Gene ontology enrichment analysis for genes upregulated or downregulated in both ST and TT conditions. Representative enriched biological processes are indicated, and the full GO enrichment results are provided in **Supplementary Table 2**. Data representative of 2 pooled independent experiments.

To further evaluate the effect of thermoneutrality on the inflammatory response to parasitic agents, we have conducted RNA-seq on the cells recruited to the peritoneal cavity 6 hours following injection with nHZ, *L. major*, or PBS as control (**Figure 5**). A principal component analysis performed on the normalized gene expression shows that the main transcriptomic differences (PC1) are caused by the type in parasitic agent injected (**Figure 5A**). A differential gene expression analysis performed between PBS-injected groups, show no difference (**Supplementary Table 2**). Likewise, when comparing the ST and TT groups for nHZ or *L. major* injected mice, we do not observe significant differences (**Supplementary Figure 9A**). These results agree with the similar recruitment of immune cells and release of cytokines and chemokines at both housing temperatures (**Figures 1B** and **Supplementary Figure 4**). As we previously published (46), peritoneal infection with *L. major* induces a strong proinflammatory response (**Supplementary Figures 9B, C**) that is characterize by an increase in genes related to gene ontology (GO) terms related to response to stress, defense response, cytokine production and signaling, cell death, cell mobility and leucocyte activation (complete results of GO enrichment provided in **Supplementary Table 3**). Interestingly, nHZ drives a similar inflammatory response but with lower amplitude with 398 genes significantly upregulated in comparison to PBS in both ST and TT, while 293 genes have a lower expression (**Figures 5B–D**). Altogether, the transcriptomics results confirm that the housing temperature does not have a major impact in the inflammatory response observed shortly (6 hours) following IP injection of parasitic agents.

### 3.6 PbA infected mice at TT show a difference in metabolism

**Figure 6:**
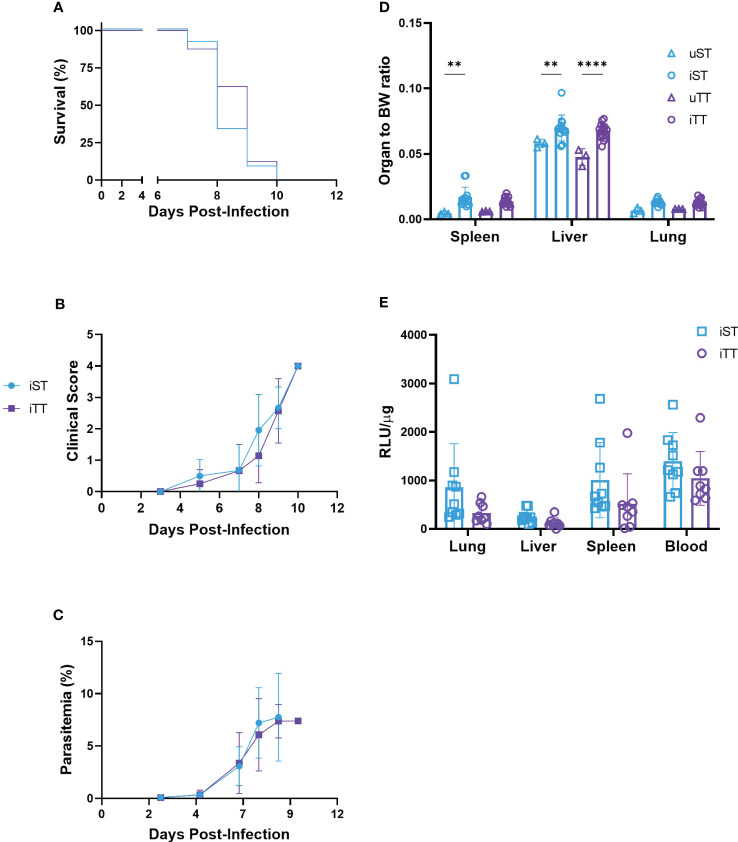
Cerebral malaria phenotypes in thermoneutral (TT) and sub-optimal temperature (ST)-housed mice. **(A)** Kaplan-Meier survival curve, **(B)** clinical score, **(C)** parasitemia, **(D)** organ to body weight-ratios, and **(E)** organ and blood parasite load (n= 8 or 9) were measured in mice infected with 10^4^ luciferase-tagged *Plasmodium berghei* ANKA-infected RBCs at TT (iTT; n=16) or ST (iST; n=13) or PBS as a mock control at TT (uTT; n=3) or ST (uST; n=3). Each experimental cerebral malaria symptom (i.e. ruffled fur, hunching, wobbly gait, limb paralysis, convulsions, and coma) contributed as one point towards the clinical score in **(B)** and mice were euthanized when score reached 4 or above. Vertical bars represent mean +/-SEM. For **(A)** statistical log-rank (Mantel-Cox) test, and for **(B–E)** mixed effects model followed by Sidak’s multiple comparison test was utilized. **** indicates p<0.0001, ** indicate p<0.01. Significance bars between iST and uTT in the liver and spleen in **(D)** are not shown for conciseness. Data is representative of 3 **(A–D)** or 2 **(E)** pooled independent experiments.

To determine the effect of thermoneutrality on the clinical progression of severe malaria, mice were acclimatized at TT and ST, infected with luciferase-tagged PbA and parasitemia was monitored for 7 to 10 days, the time for fatality to occur in this model (16). Blood and organs were collected at euthanasia (i.e. when mice reached a clinical score of 4 or above) to assess parasite loads as quantified by luciferase levels. The mice housed at TT had a similar level of parasitemia as their ST-housed counterparts, clinical progression and survival were indistinguishable (**Figures 6A–C**). In addition to blood parasite loads, for a similar organ to body-weight ratio upon euthanasia, the mice housed at TT had similar sequestration of parasite iRBCs in the lung, liver, and spleen (**Figures 6D, E**). We included results of a luciferase assay comparing infected and uninfected samples to validate use of luciferase levels to quantify parasite-load (**Supplementary Figure 10**).

Next, we monitored the serum cytokine levels of mice housed at TT and ST upon euthanasia. The choice of cytokines and chemokines measured was based on their reported implication in severe malaria (i.e. IFNγ, TNF-α, IL6, IL-1β, RANTES, MIP-1, MCP-1, IP10) (15, 60, 61). The levels of cytokines were similar in infected and uninfected animals at ST and TT (**Supplementary Figure 11**).

**Figure 7:**
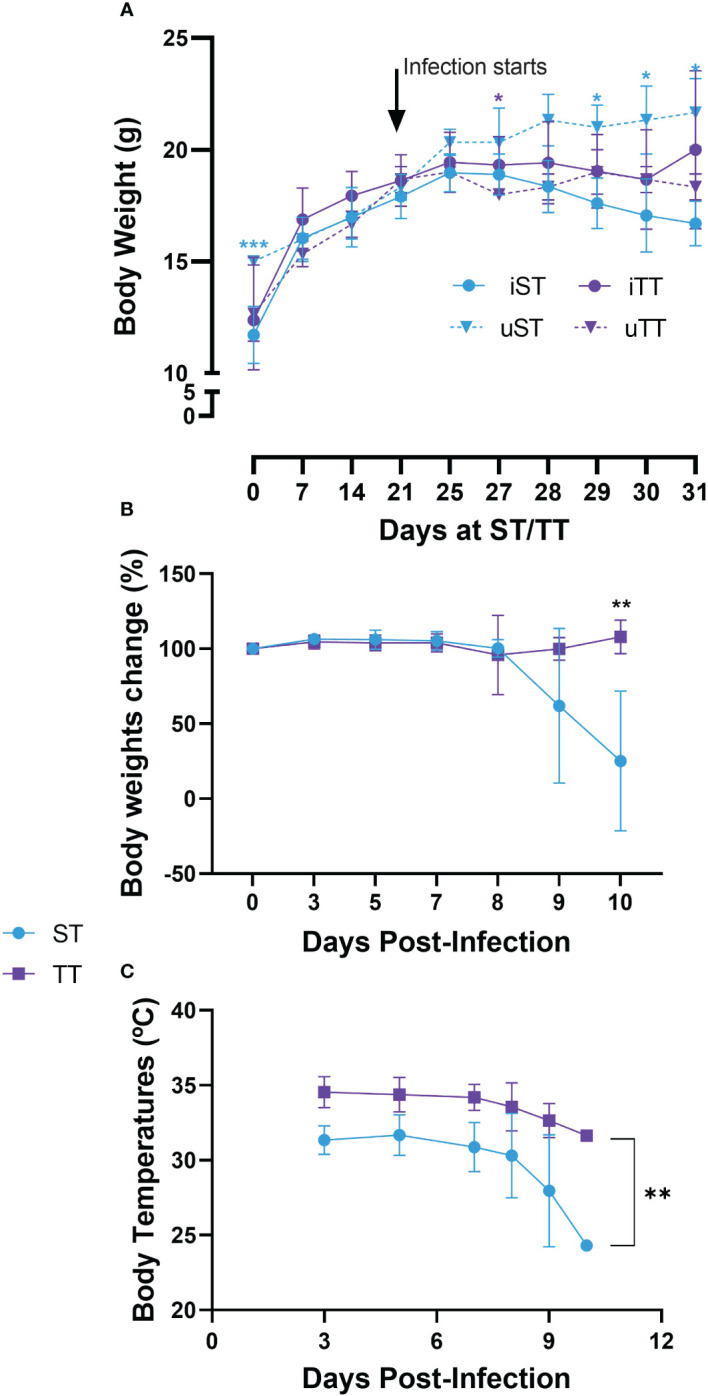
Mice at thermoneutral temperatures (TT) showed a decrease in metabolism-associated symptoms of malaria compared to suboptimal temperature (ST)-housed counterparts. **(A)** body weights, **(B)** body weight change attributable to malaria infection and **(C)** body temperature evolution of mice infected with 10^4^ luciferase-tagged *Plasmodium berghei* ANKA-infected RBCs at TT (iTT; n=16) or ST (iST; n=13) or PBS as a mock control at TT (uTT; n=3) or ST (uST; n=3) were measured. Vertical bars represent mean+/- SEM. Mixed effects models followed by Sidak’s multiple comparison test were utilized. In **(A)**, the increase from day 30 (18g) today 31 (20g) is because the two remaining mice in the trial on the last day were of higher weight than the average of the day before. Blue asterisks represent a significant change between mice in the iST and uST groups. Purple asterisks represent a significant change between mice in the iTT and uTT groups. For **(B)**, the formula used to measure change in body weights was: (Weight of animal day x/weight of same animal day of infection) x 100. ** indicates a p-value of <0.01, * indicates a p-value of <0.05. Data representative of 3 pooled independent experiments.

Losses in body weight and temperature are metabolic hallmarks of mice infected with PbA (16). We confirmed an overall progressive loss of body weight in mice housed at ST after day 27 (i.e. 6 days post-infection) (**Figure 7A**) and a loss of body weight compared to the weight at the start of the infection on day 9 and 10 post-infection (**Figure 7B**). However, the weight of the infected mice housed at TT only differed from their uninfected counterparts on day 27 (i.e. 6 days post-infection), when their body weight was greater on average, and the body weight change attributable to the infection stayed relatively constant. Similarly, the body temperature of the infected mice housed at ST decreased dramatically after day 8 post-infection (**Figure 7C**). The body temperature of the mice housed at TT decreased minimally in the same time frame. Of note, infrared thermometers tend to proportionally underestimate temperatures that would be obtained from rectal thermometers by a few degrees and have greater margin of errors, which explain why the temperatures at early infection stages were lower than expected (43). Altogether, these results suggest that malarial symptoms linked to metabolism are attenuated in mice housed at thermoneutrality. These results are in line with studies conducted on other parasitic disease models where thermoneutrality was protective for disease presentation in *Leishmania* and *Trypanosoma* infections (61–63).

### 3.7 Effect on housing temperature on the life cycle of *Plasmodium berghei* ANKA

**Figure 8:**
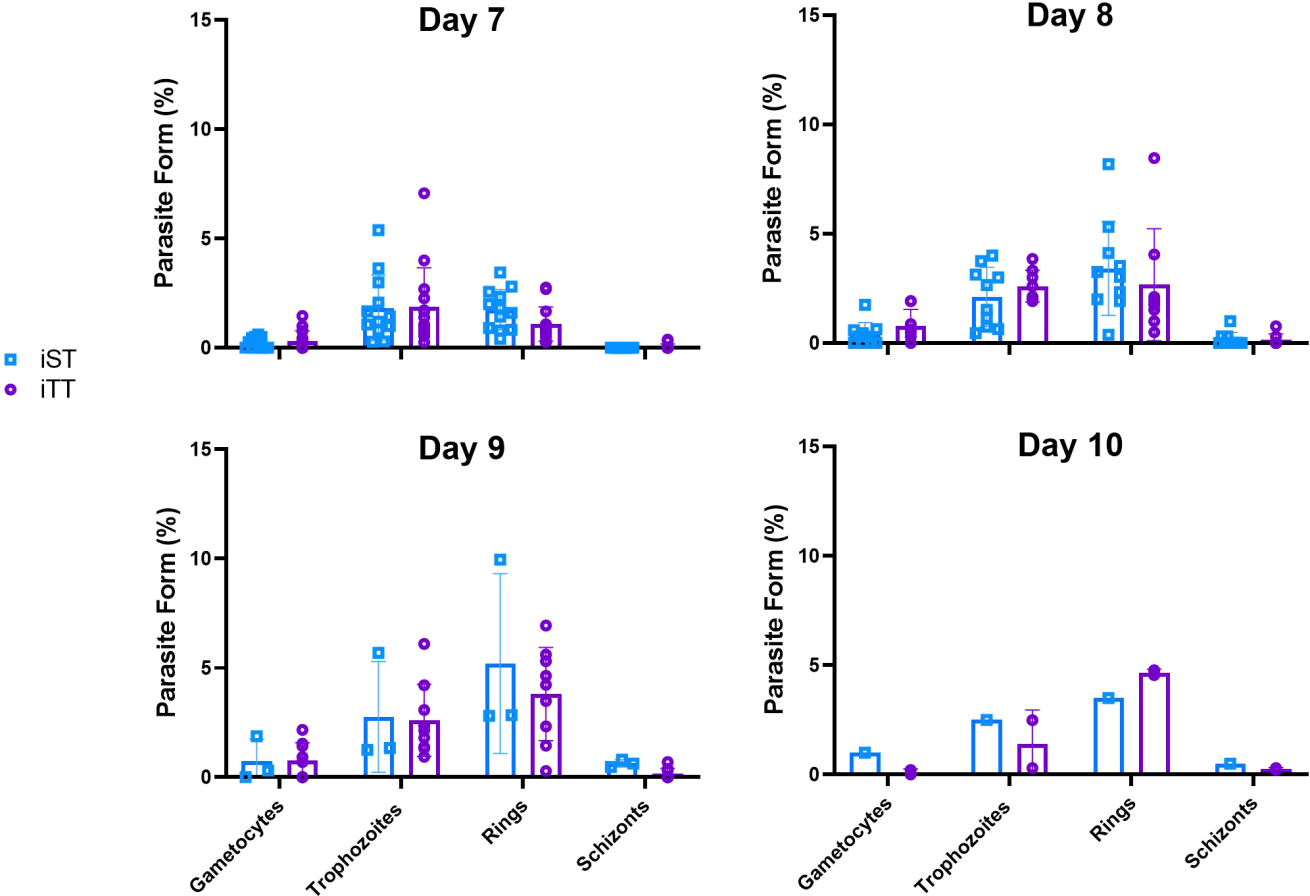
Thermoneutrality impact on parasite life-cycle stages distribution. The percentage of gametocytes, trophozoites, ring-stage and schizonts in the smears of mice infected with 10^4^ luciferase-tagged *Plasmodium berghei* ANKA-infected RBCs at thermoneutral temperature (iTT) or sub-optimal temperatures (iST) on day 7, 8, 9 and 10 after infection. Vertical bars mean+/- SEM. Two-way ANOVA followed by Sidak’s multiple comparison test was utilized. No temperature-based statistical difference was observed. Data representative of 3 pooled independent experiments.

In order to investigate whether the life cycle of the parasite is affected by thermoneutrality, we monitored the various blood life-cycle stages of parasites (i.e., gametocytes, schizonts, ring forms and trophozoites) in the blood smears of mice housed at ST and TT over the course of infection with PbA. We found that the distributions of the parasitic forms in the different stages were similar from day 7 to 10 after infection at both temperatures (**Figure 8**), suggesting that thermoneutrality did not have a direct effect on the life cycle of the *Plasmodium* parasite. This agrees with results from previous studies where the schizont size did not differ in animals housed at 28°C compared to 21°C (62). However, it must be considered that the difference in nighttime temperature may be the reason why we did not observe a difference.

## 4 Discussion

Malaria remains a global disease associated with significant mortality (1). In addition, new challenges like global warming and potential wider spread of vector mosquitoes, the COVID-19 pandemic, hurdles in malaria vaccination implementation and drug/insecticide resistance are stalling efforts to control this disease if novel solutions are not found (1). A promising avenue for disease control is new immune-based targets for therapy and/or prophylaxis. However, effective immune targeting requires the use of appropriate animal models that are representative of the human disease to ensure predictability and applicability to human trials (63). Although many of our results did not show a significant change in response to malaria at TT or ST, they are the first experiments to elucidate whether thermoneutrality influences malaria disease progression, to our knowledge. For this reason, we believe that our set of experiments constitute a first step in determining whether traditional ST-housed mice may not be representative of the metabolic condition of the host and could potentially undermine efforts to understand malaria and find effective disease control and/or therapy targets.

### 4.1 Inflammatory response to parasitic agents at thermoneutrality

Sub-optimal temperatures have been shown to impact the host immune response to diseases by decreasing the amount of energy allocated to drive the immune cell metabolism and use it for heat generation instead (21). There is also evidence that norepinephrine released by ST-housed mice may act directly on immune cells bearing β-adrenergic receptors (β-AR) and modulate their response. For example, myeloid-derived suppressor cells (MDSC) proliferate in greater number at ST in response to β-AR activation and change the outcome of cancer and host vs. graft disease progression by suppressing T cell proliferation (64, 65). Murine macrophages have been shown to be impacted by ST in various ways. Studies have shown that ST is associated with M2 type macrophage differentiation and others have shown pro-inflammatory macrophage response (i.e., MCP-1, IFNγ, TNF-α, IL-1β, IL-6, IL-4, IL-13) (49, 66, 67). By contrast, in animals at TT, macrophages produced lower amounts of these pro-inflammatory cytokines and were producing tissue-repair associated immune factors (i.e., HMGB1) (68). In addition, TT have been shown to change the inflammatory response to infectious agents and parasitic diseases (21). In rodents injected IP with bacteria or bacterial agents, thermoneutrality increased the inflammatory response with more neutrophil infiltration at TT without major changes in the release of pro-inflammatory cytokines (52, 69). In viral infections, TT has been shown to reduce pro-inflammatory cytokine levels and leukocyte infiltration (70–72). In parasitic diseases, TT may be protective by changing the inflammatory response of the host to parasitic agents. Some cells are present in greater number at the site of infection with *Trypanosoma cruzi* and *Leishmania mexicanum* (73–75). However, results describing inflammatory response to nHZ and/or *L. major* using a peritonitis model at thermoneutrality were never reported to our knowledge. In our inflammatory response study, we showed that lower amounts of inflammatory cells were recruited following *L. major* infection, in the PEC after injection with parasitic agents. Those results differ from results obtained at TT in mice injected with LPS in a peritonitis model but aligns with viral and some parasitic infection models obtained at TT (52, 69–71, 73–75). By whole transcriptome profiling, we have characterized for the first time the inflammatory response to nHZ, which leads to the recruitment of immune cells with strong pro-inflammatory transcriptional programs. However, we did not observe any major differences in the pro-inflammatory cytokine transcripts or levels by temperature. This may have been due to the limited number of samples that we obtained and/or because this is a fast response to pathogens (6 hours) which might not be sufficient time to see a difference in metabolism-induced changes to the immune system. Of note, low levels of certain pro-inflammatory cytokines (e.g. IL-1β, IL-10 and IFNγ) were unexpected but may also have been due to the changing kinetics of cytokine release overtime (8). Although we showed limited differences in results by housing temperature, we cannot rule out that thermoneutrality does not impact the inflammatory response to parasitic diseases like with other relevant mice models and our results may provide direction for further investigations.

One possible future direction would be using in depth flow cytometry to phenotype the infiltrating cells inside the PEC to unravel the roles of other cell population that might be involved in dampening the host inflammatory response at TT. For example, regulatory cells like γδT cells, MDSCs and Tregs which have all been shown to play a role in dampening the immune response to malaria might have been recruited to the PEC in different proportions during our injections at TT vs. ST (15, 76, 77). This would also help identify if any early adaptive immunity mechanisms are involved in the differential response to parasitic agents at TT compared to ST.

In addition, this study is the first one to our knowledge where the release of EVs upon parasitic IP injection is reported at TT. Traditional means of EV detection (i.e., TEM and NTA) were used as well as a novel method of EV phenotyping (i.e., hsFC) that allows for differentiation of EVs derived from specific cell types through detection of unique markers (28, 29). We showed an overall trend of lower concentration of EVs in the PEC of mice at TT vs. ST which aligns with our results of lower recruitment of inflammatory cells at the site of injection. Although we did not observe any significant temperature-based difference for each EV populations we surveyed (i.e., Leukocyte-derived, Proteasome-derived, Neutrophil-derived, Platelet-derived), we were able to show for the first time that they were released in mice PEC at both temperatures, upon injection with *L. major*. Platelets are not amongst the cells recruited to the PEC during inflammation, yet we detected the presence of platelet-derived EVs. This infiltration of platelet EVs to the site of inflammation has been shown before in rheumatoid arthritis but never in the case of murine malaria or leishmaniasis (78). These results suggest that EVs could be important modulatory factors in the inflammatory response to parasitic agents. In the future, analyzing the protein cargo of these EVs by proteomics would give further insight on the potential roles of EVs in the inflammatory response of malaria and other parasitic diseases at thermoneutrality. These methods would allow us to determine if there are any unique proteins involved in biological processes that are carried by EVs of mice housed at TT compared to ST, or if some proteins are present in greater or lower amounts at either temperature.

### 4.2 Disease progression of severe malaria at thermoneutrality

Thermoneutral temperatures have been shown to influence the disease progression and outcome in a variety of disease models (21). For some viral diseases and parasitic infections, thermoneutrality has been shown to be protective (70–73, 75). Particularly, mice infected with *T. cruzi* had an increased survivability and decreased symptoms (73). There are no studies reported on the impact of thermoneutrality on severe malaria. However, early experiments investigating the role of temperature on malaria disease evolution can give us an insight on how thermoneutrality might impact the severe malaria model (62, 79, 80). In our disease progression study, we showed that mice infected with PbA showed no change in survivability which is different from a study where *Plasmodium berghei* KBG-173-infected mice housed at different temperatures (including 27°C) had higher mortality rates including when housed individually, regardless of sex and age (80). However, in this study, mice were not acclimatized for three weeks as in ours. During our acclimatization period mice at TT gained slightly more body weight than those at ST which might have played a protective role during the disease progression as the mice did not experience as dramatic loss of a body weight and body temperature compared to their ST counterparts. While our results show that mice at TT can re-allocate the energy that would have been lost while fighting the infection at ST to continue their growth, it does not show a statistically significant greater loss in bodyweight at ST compared to TT attributable to the infection, except at the very last day post-infection where most animals had succumbed to the infection and the sample size had reduced. One future direction to truly elucidate whether this difference in metabolism is dependent from PbA infection status would be to perform these experiments with fully grown animals (8-9 weeks of age).

One limitation of these results are that we were logistically limited by the temperature-controlled cabinet which only allowed 15 mice to be housed at once and thus limited our control animals to only 3 per group.

When we investigated the cytokine levels in serum of mice at endpoint at TT and ST, we did not observe a difference between housing temperatures. However, we cannot exclude that cytokine levels were affected differentially during the progression of the infection. Thus, regular blood sampling throughout the course of the disease should be considered in future experiments to determine whether they could play a role during the disease progression.

Although we did not observe any effect of thermoneutrality on the parasite blood stage life cycle by microscopy, we cannot exclude that housing temperature might influence the *Plasmodium* parasite itself. A transcriptomics analysis of PbA-infected mice blood should be considered in the future to determine if certain parasite-specific genes associated with parasite growth would be up- or down-regulated at TT.

In addition, flow cytometry could be used to survey the immune cell populations and vasculature present in various organs, specifically the spleen and brain to elucidate whether they are influenced by housing temperature.

Finally, since the PbA-infected mouse model we used is only one of the many models used to study malaria, future experiments conducted at thermoneutrality should consider using other mouse models of malaria disease, including non-severe malaria models where immunological changes occur over greater period of time (15). For example, since C57BL/6 mice infected with *Plasmodium chabaudi* AS are a model for uncomplicated malaria, thermoneutrality could have an influence on the serum cytokine levels and inflammatory cell signature during the infection stage and either accelerate or slow the resolving phase (15).

Altogether, our results were limited in elucidating how thermoneutrality might impact the disease progression of a severe murine malaria model but provide directions for future more targeted investigations. Since the thermoneutral murine model more closely resembles the metabolic state of humans, having an idea of how malaria develops in those mice might improve the accuracy of our murine model to reflect how the disease progresses in humans. And as such, we join others from the thermoneutrality field in encouraging scientists to consider investigating vaccine candidates or targets for therapeutics in mice housed at TT in addition to those housed as ST (24, 25, 81–83). Since thermoneutral conditions are difficult to create in current animal housing, we welcome the use of nest building material to increase cage temperature (23). These strategies may offer a greater range for the metabolic states of our models to increase translatability to human trials which is important in our continued fight against this disease.

## Data availability statement

The datasets presented in this study can be found in online repositories. The RNA-Seq datasets for this study can be found in the Gene Expression Omnibus under the accession number GSE221454 "https://www.ncbi.nlm.nih.gov/geo/query/acc.cgi?acc=GSE221454".

## Ethics statement

All experiments using mice were carried out in pathogen-free housing under the Animal Use Protocol 7607 at McGill University, according to CACC Guidelines and approved by the McGill University Animal Care Committee.

## Author contributions

Overall project was designed and envisioned by MO. Inflammatory response preliminary results with sHZ were obtained by MP and MO. All other inflammatory response study experiments were performed by FV and MO. High-sensitivity flow cytometry experiments were performed by IA in the laboratory of EB, data was analyzed by IA and FV. MH and MD donated the LWA+ proteasome marker for hsFC. TEM sample preparation and imaging was performed by GD. NTA measurement was performed by GD and analysis was done by FV. The RNAseq experiment was performed by FV and analyzed by US and DL. Disease progression study experiments were performed by FV. Statistical analysis was performed by FV. DPL was consulted for questions about mice housing at thermoneutrality. FV wrote the manuscript with input from all authors. All authors contributed to the article and approved the submitted version.

## Supplementary material

Supplementary Figure 1 Real-time and average room thermoneutrality room temperatures. Temperature of room where mice were housed in a temperature-controlled cabinet at thermoneutrality (27 – 31°C) between October 11 and 27, 2020. Temperatures were higher during the day than at night to account for the fact that mice are more active at night and thus, temperature inside the cage increase.

Supplementary Figure 2 Inflammatory cells are recruited by different doses of sHZ at sub-optimal temperatures (ST) in the PEC. **(A)** Total number of inflammatory cells and **(B)** proportion of immune cell subsets recruited in synthetic hemozoin (sHZ)-treated Balb/c mice were measured after 6 hours. Balb/c mice were housed in groups of 5 and injected with 3 doses of sHZ: 100μg, 250μg and 500μg (n = 8/group). sHZ induced recruitment but not in a dose-dependent manner. Vertical bars represent +/- SEM. Two-way ANOVA followed by Sidak’s multiple comparison test was utilized.* indicates a -value < 0.05, ** indicates a p-value < 0.01, *** indicates a < 0.001. Data representative of 2 pooled independent experiments.

Supplementary Figure 3 Hemozoin (HZ) is phagocytized by macrophages and neutrophils. Percentages of **(A)** macrophages and **(B)** neutrophils containing synthetic hemozoin (sHZ) crystals were counted in the PEC of Balb/c mice injected intraperitoneally with 100μg (n = 8), 250μg (n=8) and 500μg (n=8) at ST. sHZ were phagocytized but not in a dose-dependent manner. Vertical bars represent +/- SEM. Two-way ANOVA followed by Sidak’s multiple comparison test was utilized. **** indicates a < 0.0001, ns indicates non-significance using a two-way ANOVA with Tukey’s multiple comparison test. Data representative of 2 pooled independent experiments.

Supplementary Figure 4 Pro-inflammatory cytokines and chemokines following parasitic agent injection at thermoneutral (TT) and sub-optimal temperatures (ST). Pro-inflammatory cytokines and chemokines concentrations were monitored in the PEC of mice injected intraperitoneally with 1 mg of native hemozoin (nHZ) or 10^8^ *L. major* promastigotes at ST and TT (n = 3 to 6). Vertical bars represent mean +/- SEM. Two-way ANOVA followed by Sidak’s multiple comparison test was utilized. No temperature-based statistical difference was observed. Except for IL-1β which only had detectable levels in one experiment, data is representative of 2 pooled independent experiments.

Supplementary Figure 5 Size and concentration distribution of extracellular vesicles (EV) release is modulated by hemozoin (HZ) injection at sub-optimal temperature (ST). EV distribution and concentration **(A)**, and mean number per particle diameter **(B)** was determined by nano-particle tracking analysis from PEC lavages of Balb/c mice =4) injected intraperitoneally with 100μg, 250 μg or 500 μg of synthetic hemozoin (sHz). Data in **(A)** is representative of 2 pooled independent experiments. Data in **(B)** is a mean of 3 sequential videos of the same experiment (upper graph) or 2 independent experiments (lower graph) sequential videos.

Supplementary Figure 6 Thermoneutrality does not seem to influence the profile of certain host cell extracellular vesicle (EV)-release by parasitic agents. **(A)** Total (AV+), **(B)** leukocyte-derived (CD45+) and **(C)** Neutrophil-derived (Ly6G+) EVs in the PEC of mice injected intraperitoneally with 1 mg of native hemozoin (nHZ) or 10^8^ *L. major* promastigotes were monitored by high-sensitivity flow cytometry at thermoneutral (TT) (n= 5 or 6) or sub-optimal temperatures (ST) (n = 5 or 6). Vertical bars represent mean +/- SEM. Two-way ANOVA followed by Sidak’s multiple comparison test was utilized. * indicates a p-value < 0.05, *** indicates a p-value< 0.001, **** indicates a p-value < 0.0001. Significance bars between Control ST and *L. major* TT, nHZ ST and *L. major* TT, Control TT and *L. major* ST, and, nHZ TT and *L.major* ST in **(A-C)** are not shown for conciseness. Bar graphs data representative of 2 pooled independent experiments. Scatter plots illustrate results for single representative mice injected with PBS and *L. major*.

Supplementary Figure 7 Fluorescence minus one (FMO) and isotype staining controls for EV detection by high-sensitivity flow-cytometry of representative samples. FMO and isotype antibody mixes were were incubated with aliquots of PEC lavages of mice injected intraperitoneally with 1 mg of native hemozoin (nHZ) or 10^8^ *L. major* promastigotes at sub-optimal and thermoneutral temperatures. Scatter plots illustrate results for a single representative mouse injected with *L. major* at ST and show high specificity of the antibody-panel used.

Supplementary Figure 8 Negative controls for EV detection by high-sensitivity flow-cytometry of representative samples. **(A)** Total (AV+), **(B)** leukocyte-derived (CD45+), **(C)** Neutrophil-derived (Ly6G+) **(D)** Platelet-derived (CD41+), **(E)** Proteosome-derived (AV+LWA+CT+) EVs percentages in PEC samples with or without detergent treatment. Markers were incubated with aliquots of PEC lavages of mice injected intraperitoneally with 10^8^ *L. major* promastigotes and treated with Triton X-100 to lyse EV membrane **(A–E)** and EDTA **(A, D)** to lyse Annexin V (n=3 or 5). The treatment with detergent dramatically decreased the percentage of detected EVs. Bars represent mean +/- SEM.

Supplementary Figure 9 Transcriptome profiling of peritoneal cells following parasitic agent injections. The transcriptome of peritoneal cells from mice housed at sub-optimal (ST; n=5 or 6) or thermoneutral temperature (TT; n=5 or 6) and injected with either PBS, 1 mg of native hemozoin (nHZ), or 10^8^ *L. major* (Lm) promastigotes was assessed by RNA-seq. **(A)** Volcano plot for the differential expression analyses comparing the Lm ST to Lm TT and nHZ ST to nHZ TT. **(B)** Volcano plot for the differential expression analyses comparing the Lm groups at ST and TT to their respective control PBS groups. The full DGE results are provided in **Supplementary Table 1**. **(C)** Plot comparing the fold change of gene expression in response to Lm injection between ST and TT conditions. Orange dots represent genes with significant (FDR < 0.001) change in both ST and TT conditions. Data representative of 2 pooled independent experiments.

Supplementary Figure 10 Results of luciferase assay on organs and blood of one PbA-infected mouse at sub-optimal temperature (iST) and an uninfected mouse at thermoneutral temperature (uTT). The negligeable amount of luciferase (RLU/µg) detected in the uninfected mouse at TT demonstrates the validity of our luciferase assay to detect parasite load.

Supplementary Figure 11 Cytokine levels upon euthanasia at TT compared to ST. Cytokine and chemokine serum concentrations were measured from mice infected with 10^4^ luciferase-tagged PbA iRBCs at TT (n=12) or ST (n=13) or PBS as a mock control at TT (n=3) or ST (n=3). Vertical bars represent mean +/- SEM. Two-way ANOVA followed by Sidak’s multiple comparison test was utilized. No temperature-based statistical difference was observed. Data representative of 3 pooled independent experiments.

Supplementary Table 1 Mouse monitoring data for mice at TT and ST during acclimatization phase. Cabinet temperature, cage location in cabinet, and cage temperature were monitored twice a week and food/water intake and body weights were monitored once per week during a three-week acclimatization phase. By monitoring cage temperatures twice per week at different time points, we ensured that animals were always exposed to the temperature of their assigned exposure group.

Supplementary Table 2 RNAseq differential expression analysis for peritoneal cavity cells recovered following intra-peritoneal injection of C57B6 mice housed at ST or TT with PBS, nHZ, or *L. major*. The transcriptome of peritoneal cells from mice housed at sub-optimal (ST) or thermoneutral temperature (TT) and injected with either PBS, 1 mg of native hemozoin (nHZ), or 10^8^ L. major (Lm) promastigotes was assessed by RNA-seq.

Supplementary Table 3 Gene Ontology enrichment analysis for genes upregulated following nHZ injection in both ST and TT conditions. The transcriptome of peritoneal cells from mice housed at sub-optimal (ST) or thermoneutral temperature (TT) and injected with either PBS, 1 mg of native hemozoin (nHZ), or 10^8^ L. major (Lm) promastigotes was assessed by RNA-seq.
