# Supplementary figures and images for: Thermoneutrality and severe malaria: investigating the effect of warmer environmental temperatures on the inflammatory response and disease progression
Source: Front Immunol. 2023 Jun 7;14:1128466. doi: 10.3389/fimmu.2023.1128466 (PMC10283000; doi:10.3389/fimmu.2023.1128466)

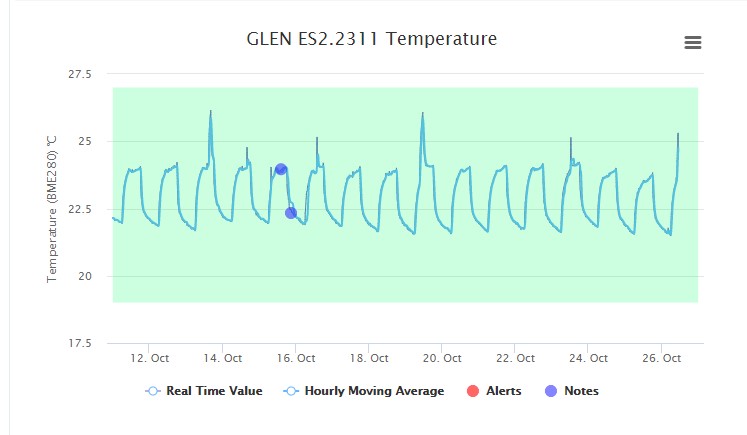

Supplement: Supplementary Figure 1 — Real-time and average room thermoneutrality room temperatures. Temperature of room where mice were housed in a temperature-controlled cabinet at thermoneutrality (27 – 31°C) between October 11 and 27, 2020. Temperatures were higher during the day than at night to account for the fact that mice are more active at night and thus, temperature inside the cage increase. [file Image_1.jpeg]

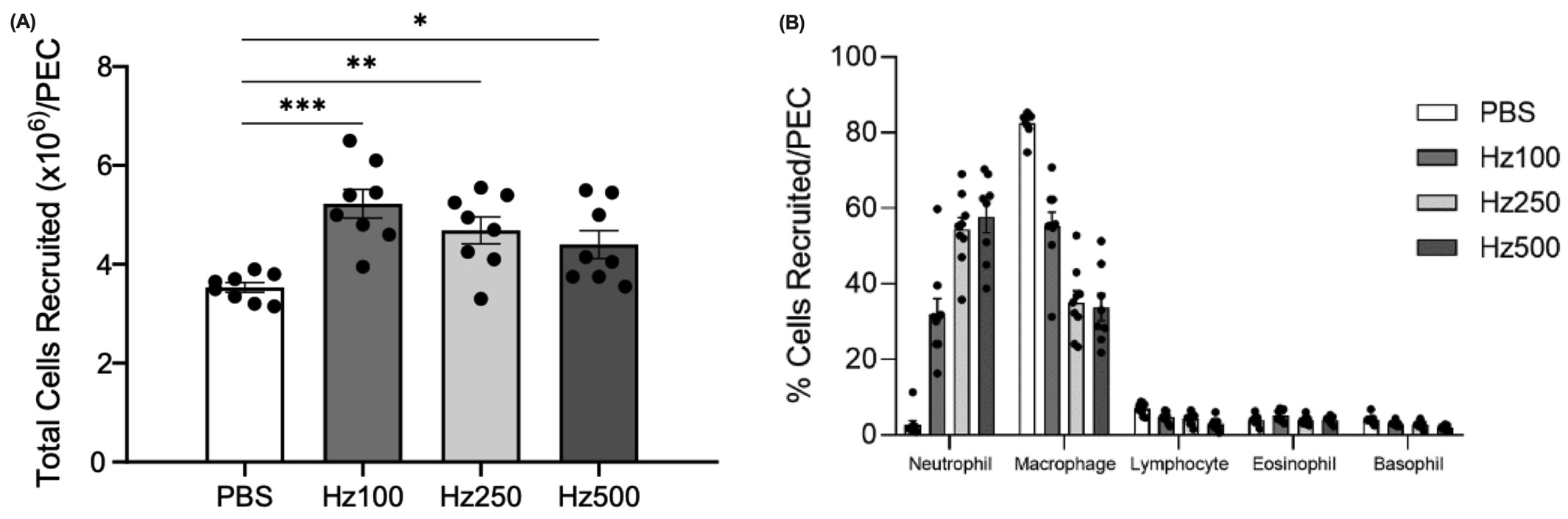

Supplement: Supplementary Figure 2 — Inflammatory cells are recruited by different doses of sHZ at sub-optimal temperatures (ST) in the PEC. (A) Total number of inflammatory cells and (B) proportion of immune cell subsets recruited in synthetic hemozoin (sHZ)-treated Balb/c mice were measured after 6 hours. Balb/c mice were housed in groups of 5 and injected with 3 doses of sHZ: 100μg, 250μg and 500μg (n = 8/group). sHZ induced recruitment but not in a dose-dependent manner. Vertical bars represent +/- SEM. Two-way ANOVA followed by Sidak’s multiple comparison test was utilized.* indicates a -value < 0.05, ** indicates a p-value < 0.01, *** indicates a < 0.001. Data representative of 2 pooled independent experiments. [file Image_2.jpeg]

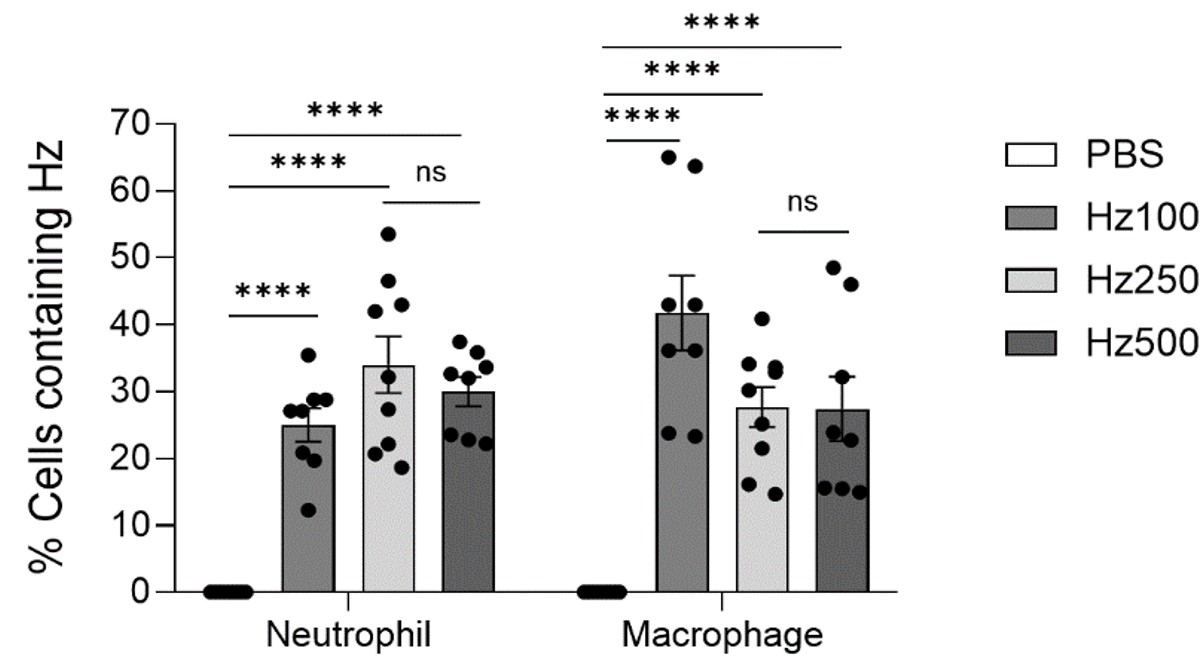

Supplement: Supplementary Figure 3 — Hemozoin (HZ) is phagocytized by macrophages and neutrophils. Percentages of (A) macrophages and (B) neutrophils containing synthetic hemozoin (sHZ) crystals were counted in the PEC of Balb/c mice injected intraperitoneally with 100μg (n = 8), 250μg (n=8) and 500μg (n=8) at ST. sHZ were phagocytized but not in a dose-dependent manner. Vertical bars represent +/- SEM. Two-way ANOVA followed by Sidak’s multiple comparison test was utilized. **** indicates a < 0.0001, ns indicates non-significance using a two-way ANOVA with Tukey’s multiple comparison test. Data representative of 2 pooled independent experiments. [file Image_3.jpeg]

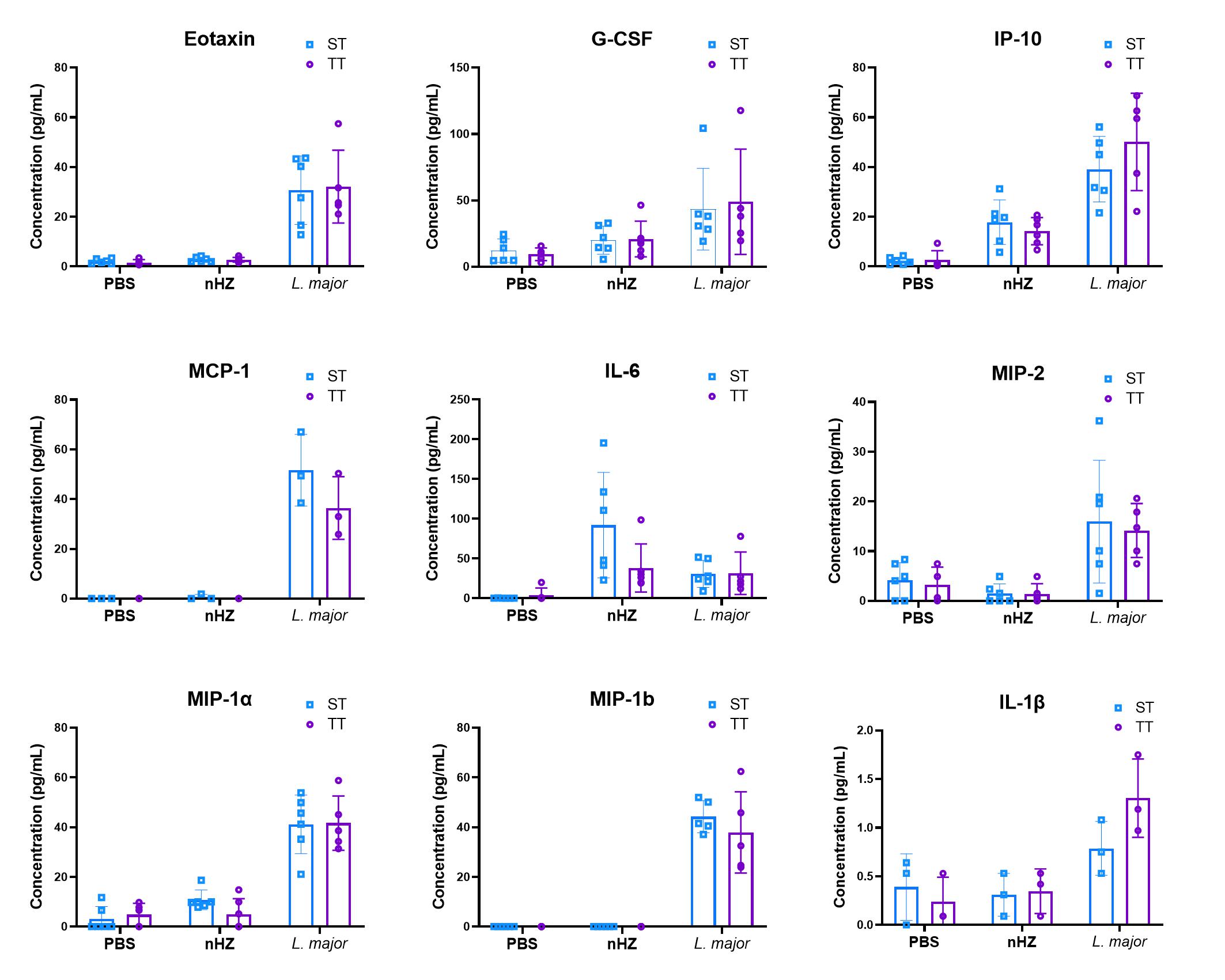

Supplement: Supplementary Figure 4 — Pro-inflammatory cytokines and chemokines following parasitic agent injection at thermoneutral (TT) and sub-optimal temperatures (ST). Pro-inflammatory cytokines and chemokines concentrations were monitored in the PEC of mice injected intraperitoneally with 1 mg of native hemozoin (nHZ) or 108 L. major promastigotes at ST and TT (n = 3 to 6). Vertical bars represent mean +/- SEM. Two-way ANOVA followed by Sidak’s multiple comparison test was utilized. No temperature-based statistical difference was observed. Except for IL-1β which only had detectable levels in one experiment, data is representative of 2 pooled independent experiments. [file Image_4.jpeg]

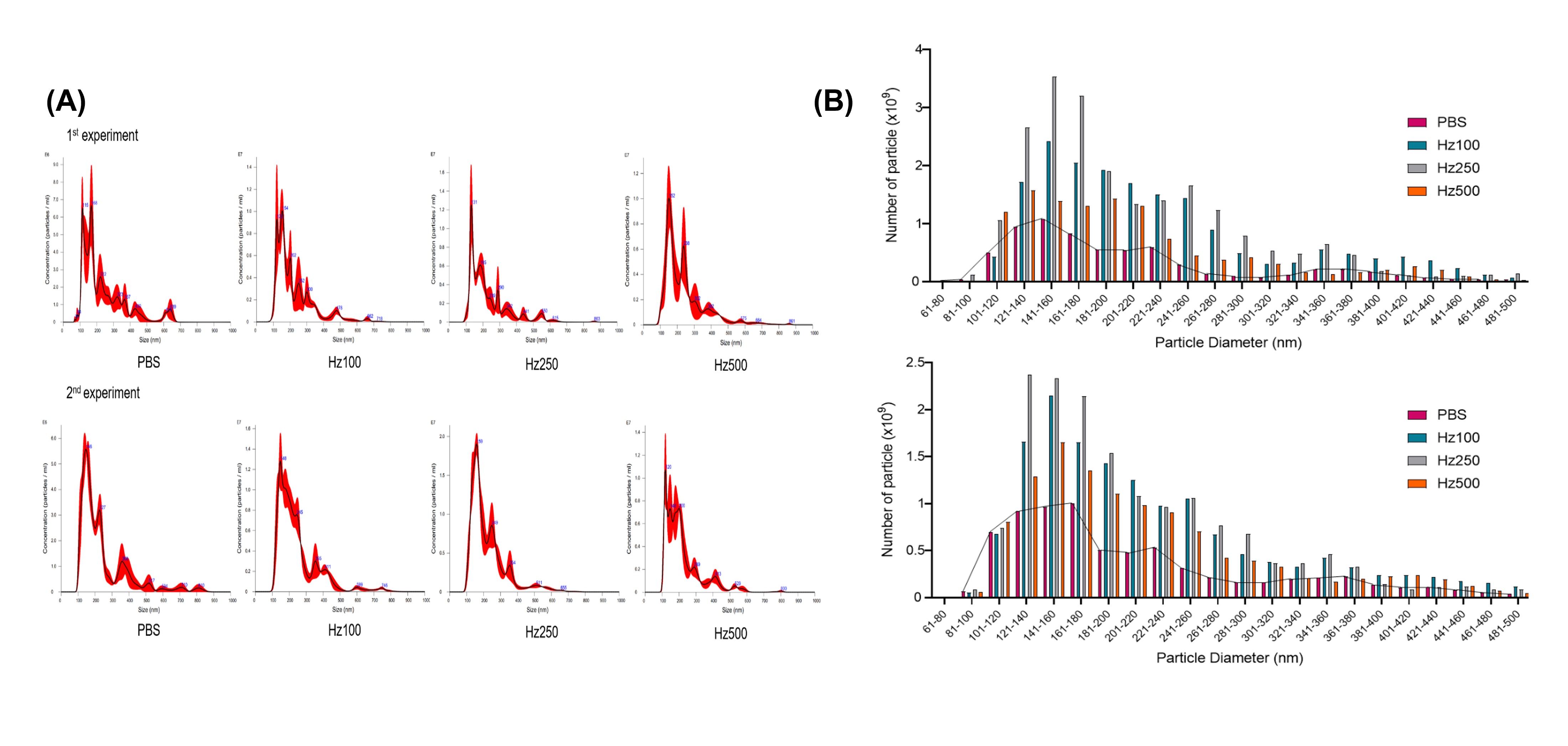

Supplement: Supplementary Figure 5 — Size and concentration distribution of extracellular vesicles (EV) release is modulated by hemozoin (HZ) injection at sub-optimal temperature (ST). EV distribution and concentration (A), and mean number per particle diameter (B) was determined by nano-particle tracking analysis from PEC lavages of Balb/c mice =4) injected intraperitoneally with 100μg, 250 μg or 500 μg of synthetic hemozoin (sHz). Data in (A) is representative of 2 pooled independent experiments. Data in (B) is a mean of 3 sequential videos of the same experiment (upper graph) or 2 independent experiments (lower graph) sequential videos. [file Image_5.jpeg]

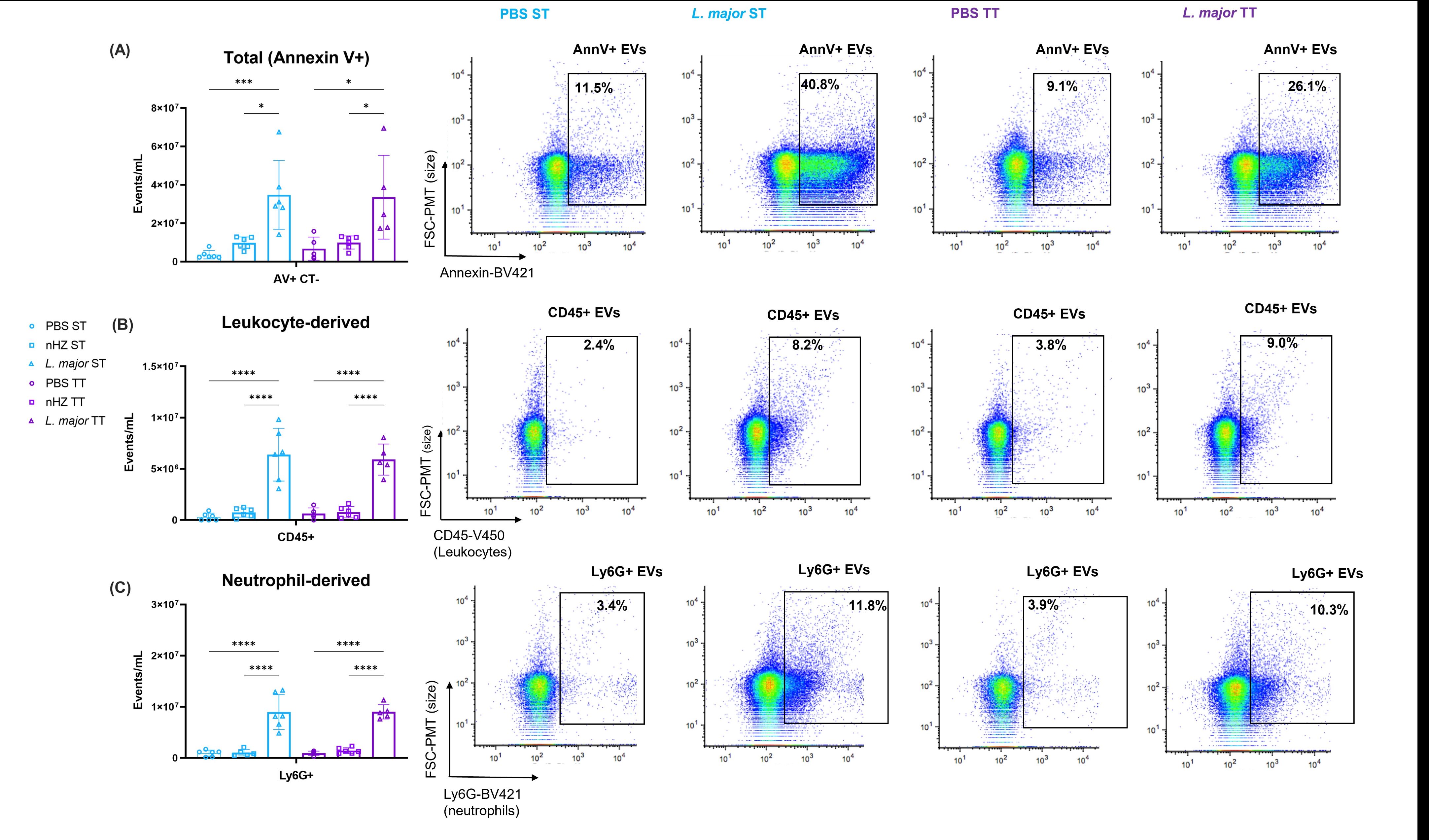

Supplement: Supplementary Figure 6 — Thermoneutrality does not seem to influence the profile of certain host cell extracellular vesicle (EV)-release by parasitic agents. (A) Total (AV+), (B) leukocyte-derived (CD45+) and (C) Neutrophil-derived (Ly6G+) EVs in the PEC of mice injected intraperitoneally with 1 mg of native hemozoin (nHZ) or 108 L. major promastigotes were monitored by high-sensitivity flow cytometry at thermoneutral (TT) (n= 5 or 6) or sub-optimal temperatures (ST) (n = 5 or 6). Vertical bars represent mean +/- SEM. Two-way ANOVA followed by Sidak’s multiple comparison test was utilized. * indicates a p-value < 0.05, *** indicates a p-value< 0.001, **** indicates a p-value < 0.0001. Significance bars between Control ST and L. major TT, nHZ ST and L. major TT, Control TT and L. major ST, and, nHZ TT and L.major ST in (A-C) are not shown for conciseness. Bar graphs data representative of 2 pooled independent experiments. Scatter plots illustrate results for single representative mice injected with PBS and L. major. [file Image_6.jpeg]

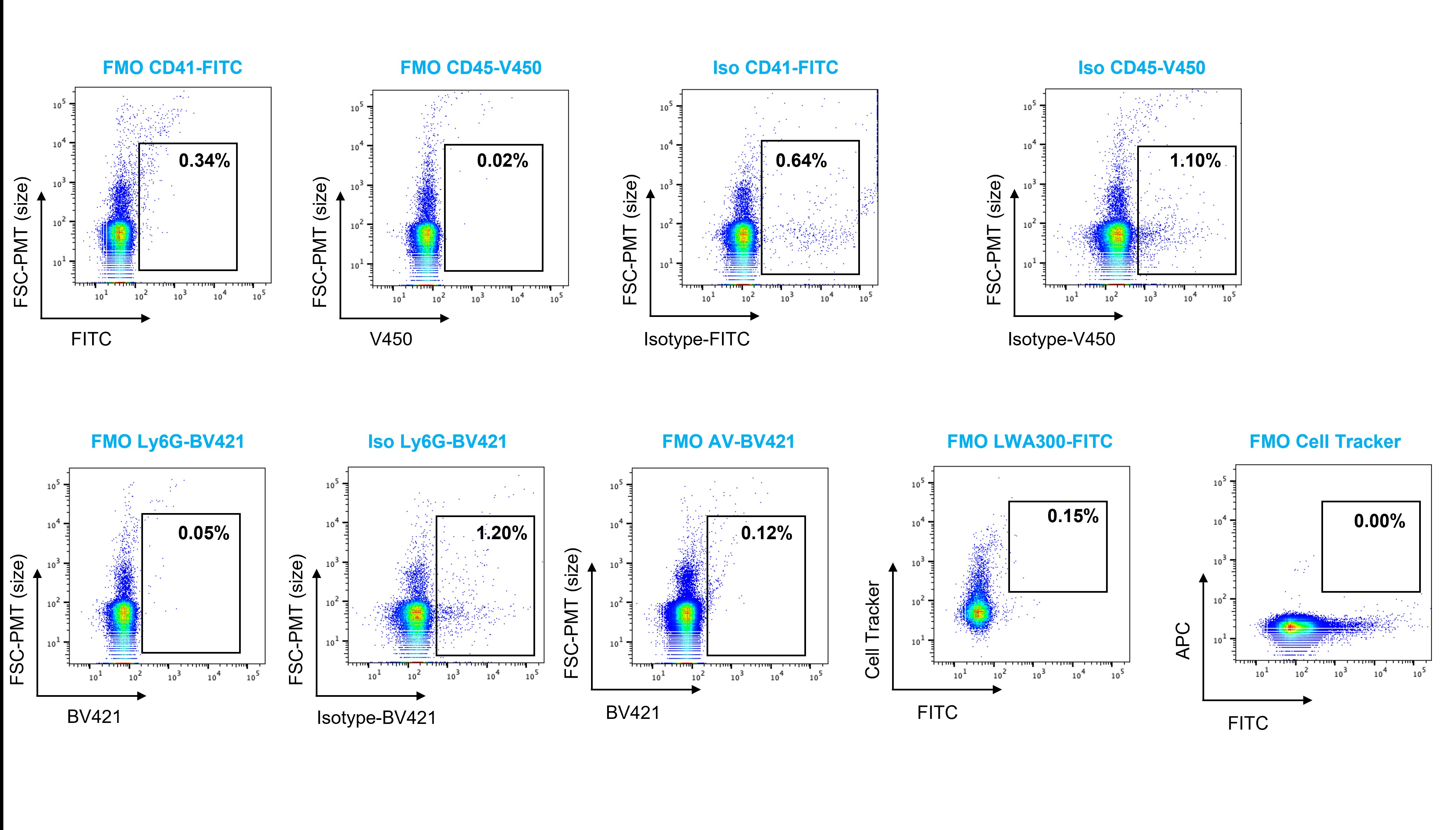

Supplement: Supplementary Figure 7 — Fluorescence minus one (FMO) and isotype staining controls for EV detection by high-sensitivity flow-cytometry of representative samples. FMO and isotype antibody mixes were were incubated with aliquots of PEC lavages of mice injected intraperitoneally with 1 mg of native hemozoin (nHZ) or 108 L. major promastigotes at sub-optimal and thermoneutral temperatures. Scatter plots illustrate results for a single representative mouse injected with L. major at ST and show high specificity of the antibody-panel used. [file Image_7.jpeg]

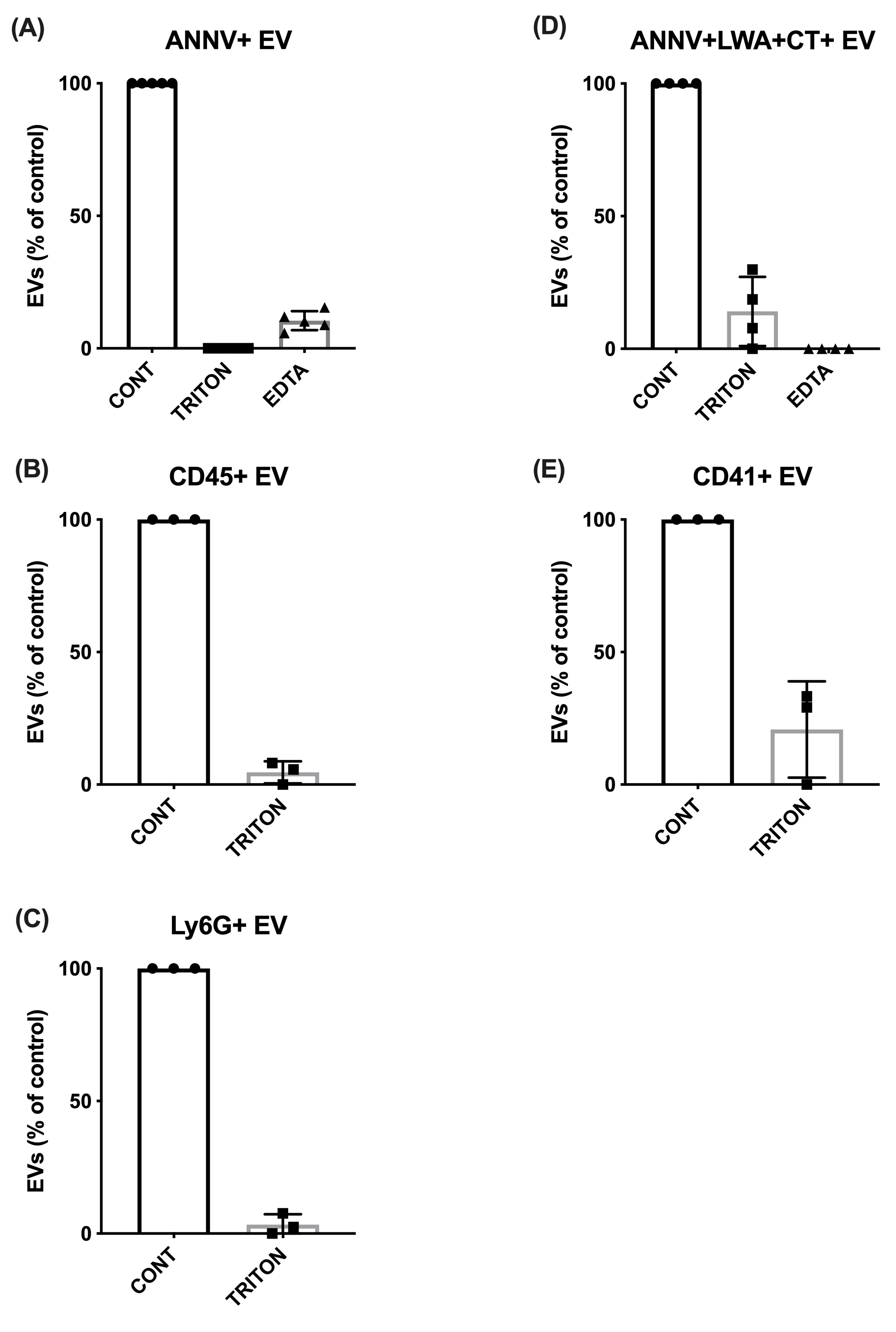

Supplement: Supplementary Figure 8 — Negative controls for EV detection by high-sensitivity flow-cytometry of representative samples. (A) Total (AV+), (B) leukocyte-derived (CD45+), (C) Neutrophil-derived (Ly6G+) (D) Platelet-derived (CD41+), (E) Proteosome-derived (AV+LWA+CT+) EVs percentages in PEC samples with or without detergent treatment. Markers were incubated with aliquots of PEC lavages of mice injected intraperitoneally with 108 L. major promastigotes and treated with Triton X-100 to lyse EV membrane (A–E) and EDTA (A, D) to lyse Annexin V (n=3 or 5). The treatment with detergent dramatically decreased the percentage of detected EVs. Bars represent mean +/- SEM. [file Image_8.jpeg]

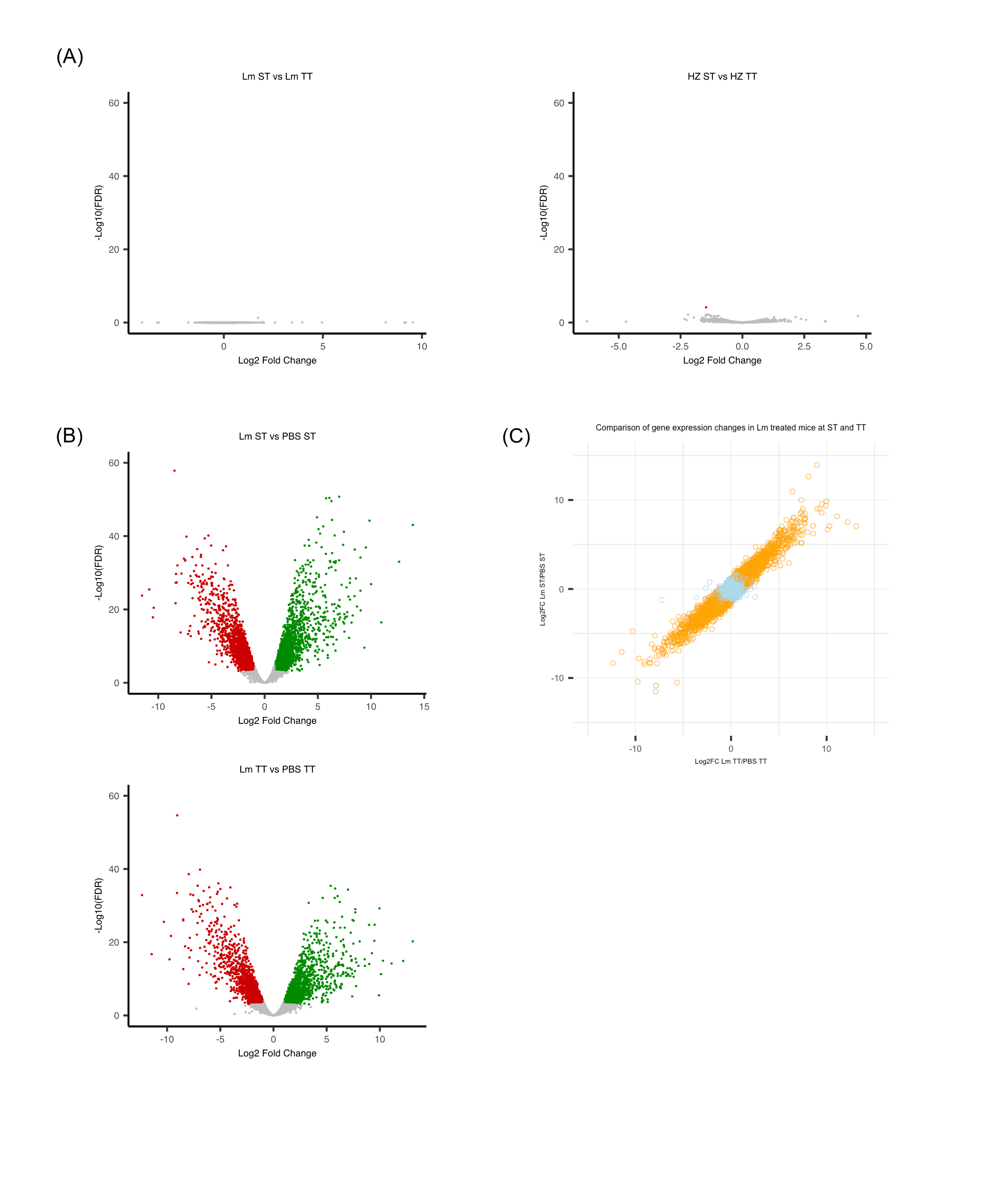

Supplement: Supplementary Figure 9 — Transcriptome profiling of peritoneal cells following parasitic agent injections. The transcriptome of peritoneal cells from mice housed at sub-optimal (ST; n=5 or 6) or thermoneutral temperature (TT; n=5 or 6) and injected with either PBS, 1 mg of native hemozoin (nHZ), or 108 L. major (Lm) promastigotes was assessed by RNA-seq. (A) Volcano plot for the differential expression analyses comparing the Lm ST to Lm TT and nHZ ST to nHZ TT. (B) Volcano plot for the differential expression analyses comparing the Lm groups at ST and TT to their respective control PBS groups. The full DGE results are provided in Supplementary Table 1 . (C) Plot comparing the fold change of gene expression in response to Lm injection between ST and TT conditions. Orange dots represent genes with significant (FDR < 0.001) change in both ST and TT conditions. Data representative of 2 pooled independent experiments. [file Image_9.jpeg]

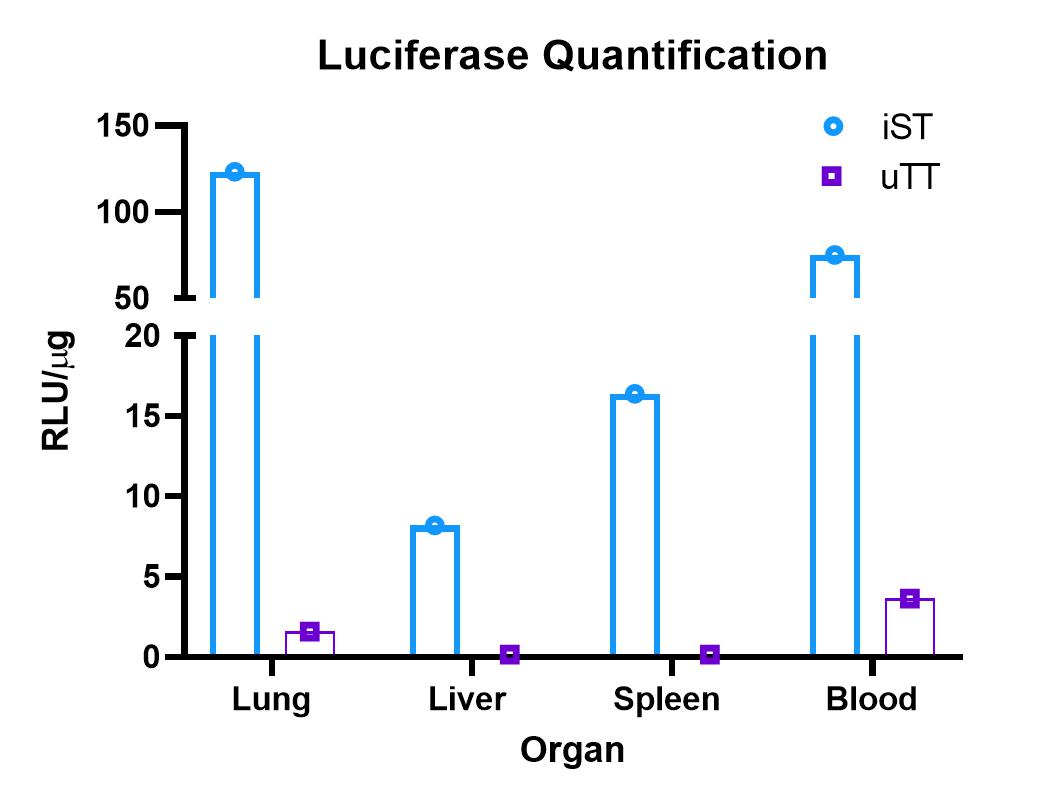

Supplement: Supplementary Figure 10 — Results of luciferase assay on organs and blood of one PbA-infected mouse at sub-optimal temperature (iST) and an uninfected mouse at thermoneutral temperature (uTT). The negligeable amount of luciferase (RLU/µg) detected in the uninfected mouse at TT demonstrates the validity of our luciferase assay to detect parasite load. [file Image_10.jpeg]

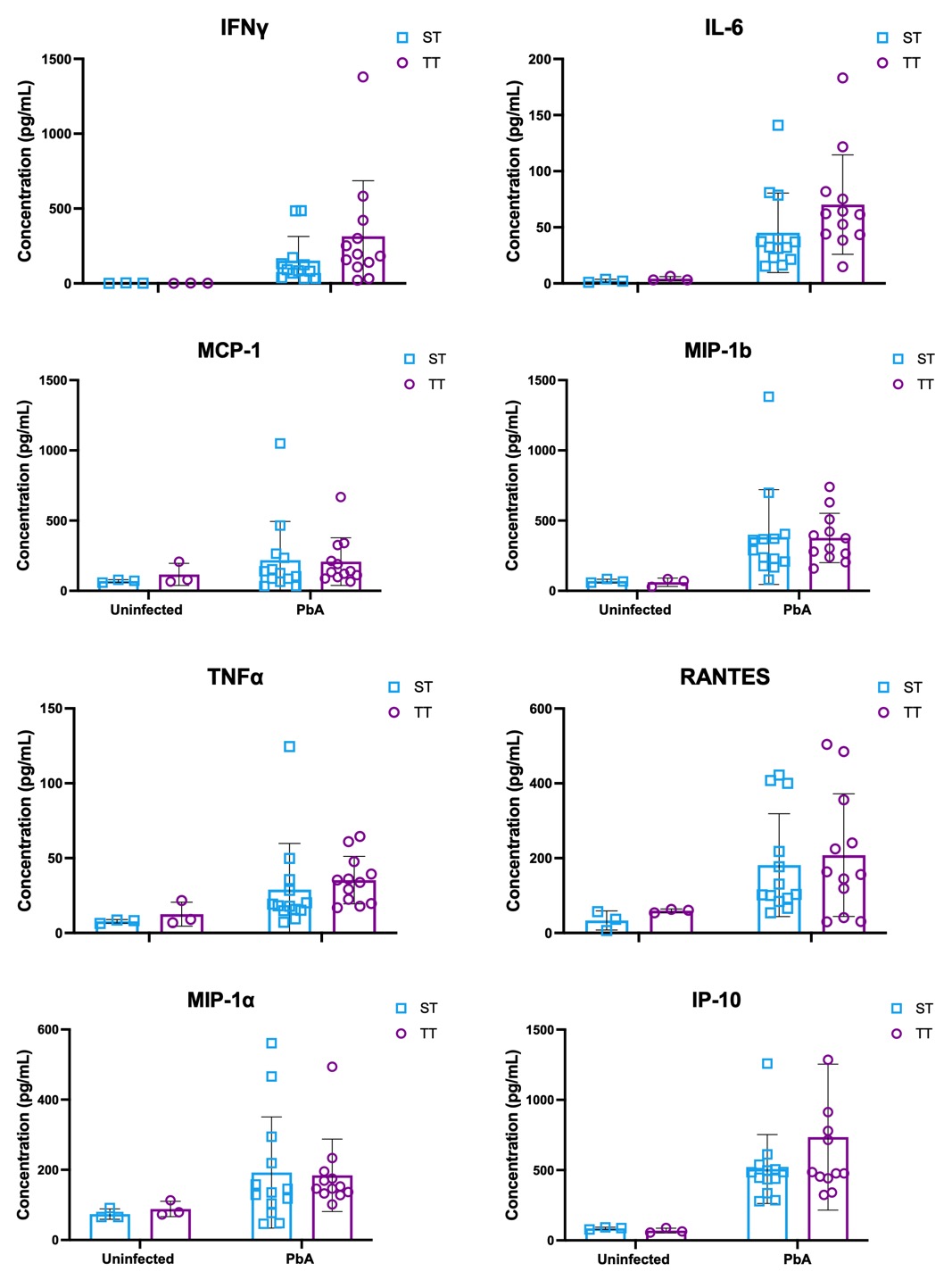

Supplement: Supplementary Figure 11 — Cytokine levels upon euthanasia at TT compared to ST. Cytokine and chemokine serum concentrations were measured from mice infected with 104 luciferase-tagged PbA iRBCs at TT (n=12) or ST (n=13) or PBS as a mock control at TT (n=3) or ST (n=3). Vertical bars represent mean +/- SEM. Two-way ANOVA followed by Sidak’s multiple comparison test was utilized. No temperature-based statistical difference was observed. Data representative of 3 pooled independent experiments. [file Image_11.jpeg]
